# Bovine Host Genetic Variation Influences Rumen Microbial Methane Production with Best Selection Criterion for Low Methane Emitting and Efficiently Feed Converting Hosts Based on Metagenomic Gene Abundance

**DOI:** 10.1371/journal.pgen.1005846

**Published:** 2016-02-18

**Authors:** Rainer Roehe, Richard J. Dewhurst, Carol-Anne Duthie, John A. Rooke, Nest McKain, Dave W. Ross, Jimmy J. Hyslop, Anthony Waterhouse, Tom C. Freeman, Mick Watson, R. John Wallace

**Affiliations:** 1 SRUC, Edinburgh, United Kingdom; 2 Rowett Institute of Nutrition and Health, University of Aberdeen, Aberdeen, United Kingdom; 3 Division of Genetics and Genomics, The Roslin Institute and R(D)SVS, University of Edinburgh, Edinburgh, United Kingdom; 4 Edinburgh Genomics, The Roslin Institute and R(D)SVS, University of Edinburgh, Edinburgh, United Kingdom; University of Bern, SWITZERLAND

## Abstract

Methane produced by methanogenic archaea in ruminants contributes significantly to anthropogenic greenhouse gas emissions. The host genetic link controlling microbial methane production is unknown and appropriate genetic selection strategies are not developed. We used sire progeny group differences to estimate the host genetic influence on rumen microbial methane production in a factorial experiment consisting of crossbred breed types and diets. Rumen metagenomic profiling was undertaken to investigate links between microbial genes and methane emissions or feed conversion efficiency. Sire progeny groups differed significantly in their methane emissions measured in respiration chambers. Ranking of the sire progeny groups based on methane emissions or relative archaeal abundance was consistent overall and within diet, suggesting that archaeal abundance in ruminal digesta is under host genetic control and can be used to genetically select animals without measuring methane directly. In the metagenomic analysis of rumen contents, we identified 3970 microbial genes of which 20 and 49 genes were significantly associated with methane emissions and feed conversion efficiency respectively. These explained 81% and 86% of the respective variation and were clustered in distinct functional gene networks. Methanogenesis genes (e.g. *mcrA* and *fmdB*) were associated with methane emissions, whilst host-microbiome cross talk genes (e.g. *TSTA3* and *FucI*) were associated with feed conversion efficiency. These results strengthen the idea that the host animal controls its own microbiota to a significant extent and open up the implementation of effective breeding strategies using rumen microbial gene abundance as a predictor for difficult-to-measure traits on a large number of hosts. Generally, the results provide a proof of principle to use the relative abundance of microbial genes in the gastrointestinal tract of different species to predict their influence on traits e.g. human metabolism, health and behaviour, as well as to understand the genetic link between host and microbiome.

## Author Summary

Methane is a highly potent greenhouse gas and ruminants are the major source of methane emissions from anthropogenic activities. Here we show in an experiment with cattle that genetic selection of low-emitting animals is a viable option based on a newly developed selection criterion. The experimental data provided a comprehensive insight into the host additive genetic influence on the microbiome, the impact of nutrition on genetics and the microbiome, and the effect of metagenomic microbial genes on the analysed traits. We developed a selection criterion to change those traits by evaluation of hosts based on the relative abundance of microbial genes. This criterion is shown to be highly informative and it is therefore suggested to be used in studies analysing different traits and species. This study provides a proof of principle that there is an additive genetic influence of the host on its microbiome and that selection for the desired host can be based on the abundance of a suite of genes in the ruminal metagenome associated with the trait. The use of this criterion will allow genetic analysis on a large number of hosts, previously a significant barrier to determination of host genetic effects on such traits.

## Introduction

By 2050, the human population will grow to over 9 billion people, and in the same time frame, global meat consumption is projected to increase by 73% [1]. However, intensive food production puts a strain on the environment, and there is a need to produce more food ethically and in a way that does not harm the environment. Methane is a greenhouse gas with a global warming potential 28-times that of carbon dioxide [2] and ruminants are the major source of methane emissions from anthropogenic activities. Finding means to mitigate methane emissions is an intractable problem, despite large international research efforts. A fundamental problem is that the ruminal microbiota is able to adapt rapidly to intervention methods that have been tried so far—such as different dietary formulations, chemical and biological feed additives, chemo-genomics and anti-methanogen vaccines [3]. In this study we show that genetic selection of low methane emitting animals is a viable option. The gut microbial ecosystem is particularly important in ruminants due to its ability to convert indigestible fibrous plant material into absorbable nutrients. From the environmental and energetic efficiency point of view, there is a disadvantage in that the anaerobic microbial fermentation process can result in excess hydrogen that is used by methanogenic archaea to produce methane and then eructed into the atmosphere. The loss of feed gross energy as methane has been estimated at 2 to 12% [4].

In order to address food security as well as economic and environmental impacts of food production, sustainable intensification has been suggested [5] with genetic improvement of feed conversion efficiency of highest importance in farm animals. Therefore, the overall aim of our work was to improve the efficiency of the rumen microbial community in converting feed into nutrients with minimal production of methane. The host animal provides the environment for the microbial ecosystem in the rumen and may therefore have an impact on its composition and efficiency. Studies in rodents and humans suggest that there is a host genetic influence on the microbiome [6–9]. In addition, research in bovine and ovine indicates that there is a host genetic influence on methane emissions and feed conversion efficiency without considering and evaluating the impact of the microbiome [10–13]. Our previous study found a phenotypic correlation between the composition of the rumen community and methane emissions [14]. However, direct evidence for a genetic control of the microbiota by the host in ruminants is rather weak. Therefore, the main aim of this study was to investigate whether there is a genetic influence of the host on the ruminal microbial community which affects methane production. If the genetics of the host animal has a significant role in determining key activities of the microbiota, then breeding would be a cost-effective tool to reduce methane emissions and improve feed conversion efficiency, provided that an accurate selection criterion is available. Therefore, this study also aimed to find the best selection criterion for mitigation or improvement of these traits.

Metagenomics allows the identification of the composition of the whole microbial community, as well as the abundance of their genes. It could be used to develop new selection criteria for difficult-to-measure traits or to understand the link between host genetics, the microbiome and its activity. Our study design allowed us to provide an insight into the genetic influence of the host animal on methane production by archaea, the impact of diets on methane emissions and their interactions with the host genetics. We found novel selection criteria related to microbial characteristics of each host which can be used to select for low methane emitting animals. Specifically, the relative abundance of microbial genes, identified in a metagenomic analysis, was highly informative for predicting methane emissions, but also for other traits such as feed conversion efficiency, and is recommended for exploitation in genetic selection of hosts or to understand the additive genetic link between host genetics and microbiome. Host selection based on a functional microbiome microarray containing microbial genes associated with methane emissions, feed conversion efficiency, health and other traits will provide a novel and cost-effective selection opportunity without measuring these difficult and costly to record traits and has the potential to enable large scale breeding for these performances. This study was carried out using cattle but the identified best microbial criterion (microbial composition, genes and pathways) to achieve insight into the host-microbiome interactions should be transferable to other traits and species.

## Results and Discussion

The results are based on a 2 × 2 factorial design experiment of crossbred breed types and diets in which methane emissions of individual animals were measured in respiration chambers and the microbial community was determined by qPCR targeting the 16S rRNA gene. The experiment was designed using sire progeny groups to estimate the genetic control of the host on methane emissions due to change in microbial community.

### Substantial variation in methane emissions and microbial community among hosts

**Fig 1:**
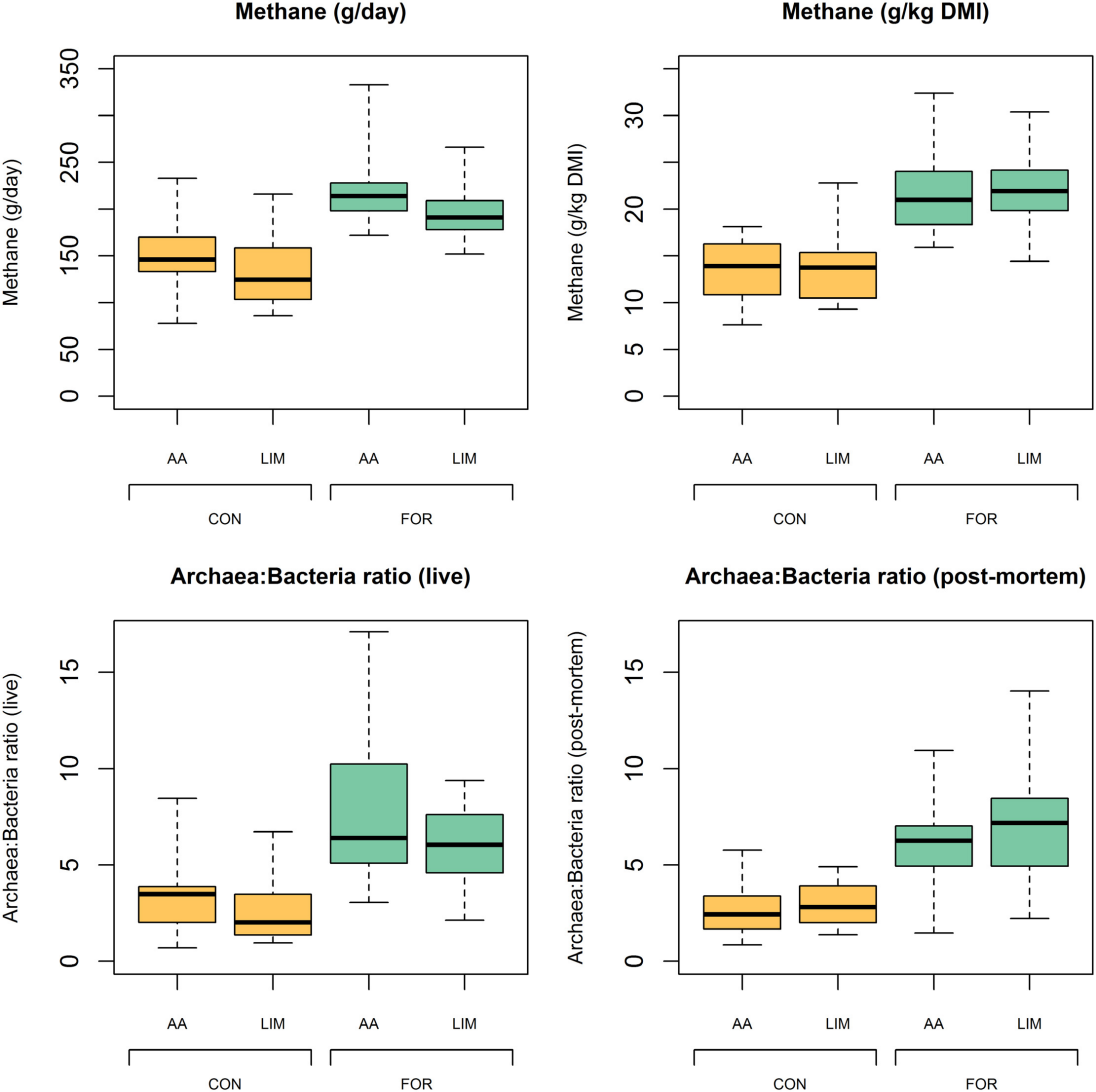
Distribution of methane emissions and archaea:bacteria ratios within breed type and diet. The box plot shows the large variation and range of methane emissions (per day or per kg DMI = dry matter intake) and archaea:bacteria ratios within crossbred breed type (AA = Aberdeen Angus sired, LIM = Limousin sired) and diet (CON = concentrate based diet, FOR = forage based diet). The total number of animals in the 2 × 2 factorial design experiment was 68.

The basis for an efficient selection program to mitigate methane emissions due change in microbial community depends on the genetic variation of these characteristics among animals. There were large phenotypic ranges in methane emissions between the extreme low and high emitting animals within breed type and diet groups (Fig 1, S1 Table and S1 Dataset). The differences in daily methane emissions between the extremes within crossbred breed type were similar between diets suggesting that the diet effect represents only a scaling effect. If at least part of the variation is influenced by the host animal, then selection for mitigation of methane emissions is expected to be efficient. Even larger variation was obtained for relative archaeal abundance measured as archaea:bacteria ratios, within breed type and diet, as shown by coefficients of variation in the range of 35% to 50% and 39% to 65% for forage and concentrate-based diets respectively.

### Breed types and diets are changing methane emissions and microbial community

A first indication for a host genetic influence on methane emissions and on the composition of the microbial community can be derived from breed type differences. The least squares means (LSM) for daily methane emissions were significantly different at 184 g/d and 164 g/d for the Aberdeen Angus (AA) and Limousin (LIM) breed types respectively, but not significantly different for methane emissions per kg dry matter intake (DMI) (Table 1). These results indicate that the significant difference between breed types in daily methane emissions were due to higher feed intake of AA (11.3 ± 0.36 kg and 10.2 ± 0.36 kg DMI for the concentrate and forage based diet, respectively) compared to LIM (9.8 ± 0.37 kg and 8.8 ± 0.36 kg, for the same diets, respectively). Animals offered the forage based diet had significantly higher methane emissions than those offered the concentrate based diet. This difference is due to higher propionate production from fermentation of starch in concentrate diets, which leads to less hydrogen being available for methanogenesis [15–17]. Estimated LSM for archaea:bacteria ratios taken from live or slaughtered animals were significantly different for diet effects, but not for breed type effects. In the interpretation of the breed type results, it has to be considered that this effect represents only an expected 2/3 of the additive genetic contribution of the sire breed and that non-additive genetic effects can also have an impact. Consistent with diet effects on methane emissions, low archaea:bacteria ratios were obtained for animals offered the concentrate- in comparison to forage-based diet. The differences in archaea:bacteria ratios between diets were 3.7 for both rumen contents samples taken from live and slaughtered animals, suggesting that these measurements can be used interchangeably.

**Table 1:** Comparison of least squares means (LSM) for the breed type and diet effects on methane emissions and archaea:bacteria ratios.

Trait	Breed type	LSM	SE	P-value	Diet	LSM	SE	P-value
Methane g/day	Aberdeen Angus sired	183.8	5.63	<0.0001	Forage	205.2	5.72	<0.0001
	Limousin sired	164.4	5.83	<0.0001	Concentrate	142.9	5.75	<0.0001
	Breed type difference	19.4		0.0196	Diet difference	62.3		<0.0001
Methane g/kg DM	Aberdeen Angus sired	17.37	0.555	<0.0001	Forage	21.63	0.566	<0.0001
	Limousin sired	17.96	0.575	<0.0001	Concentrate	13.69	0.564	<0.0001
	Breed type difference	-0.59		0.463	Diet difference	7.94		<0.0001
Archaea:Bacteria ratio in live animals	Aberdeen Angus sired	5.53	0.498	<0.0001	Forage	6.86	0.520	<0.0001
	Limousin sired	4.41	0.536	<0.0001	Concentrate	3.09	0.519	<0.0001
	Breed type difference	1.12		0.132	Diet difference	3.77		<0.0001
Archaea:Bacteria ratio in slaughtered animals	Aberdeen Angus sired	4.47	0.347	<0.0001	Forage	6.52	0.347	<0.0001
	Limousin sired	4.88	0.351	<0.0001	Concentrate	2.82	0.351	<0.0001
	Breed type difference	-0.41		0.407	Diet difference	3.70		<0.0001

### Host genetics affects methane emissions

**Fig 2:**
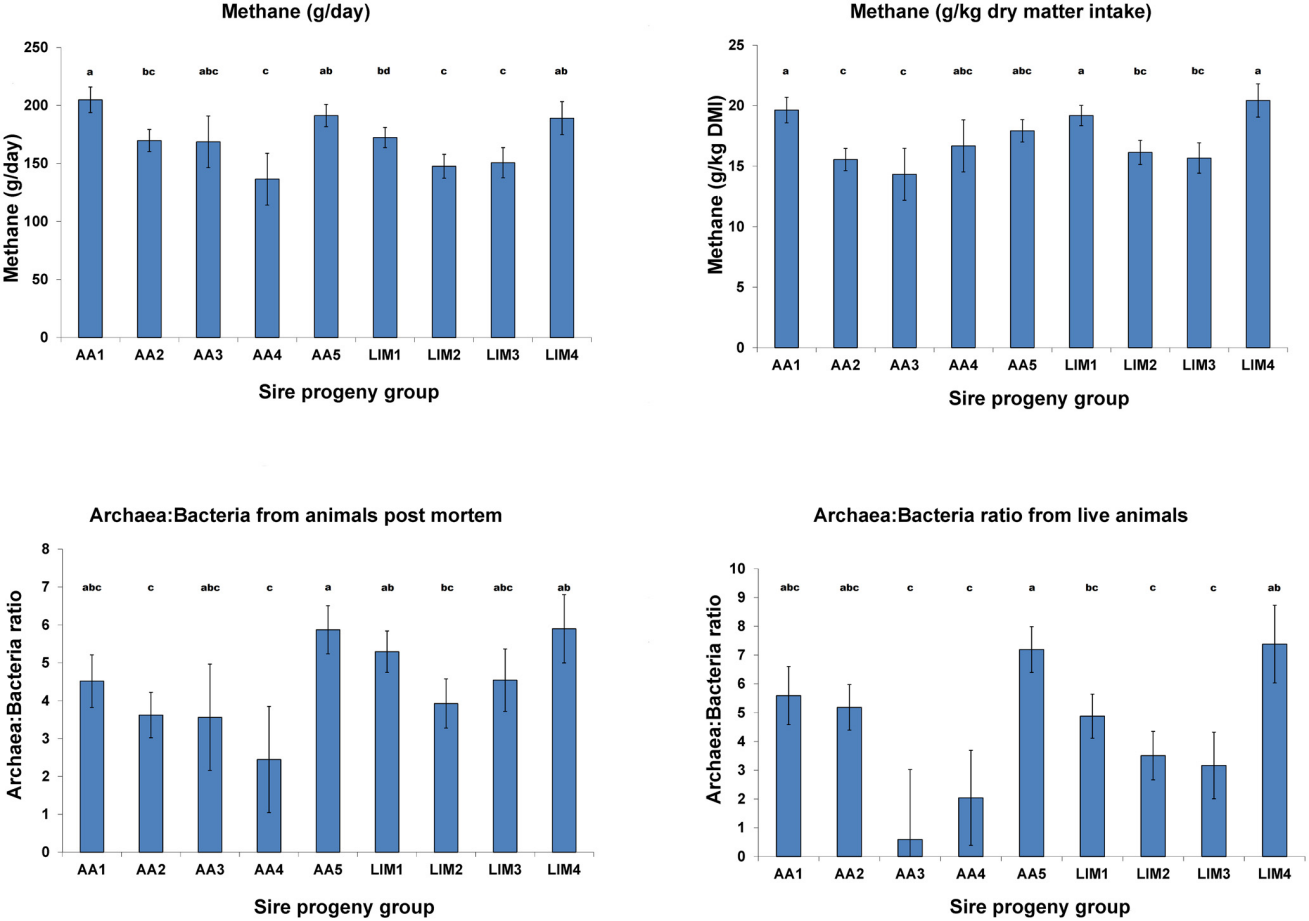
Host genetic effects on methane emissions and relative microbial abundance. Host genetic effects were estimated by least squares means (± standard errors, different letters above bars indicate significant different estimates) of sire progeny groups (AA = Aberdeen Angus sired, LIM = Limousin sired) adjusted for diet, respiration chamber and randomized block effects. Relative microbial abundance was calculated as archaea:bacteria ratio.

Sire progeny groups differences were used to identify the host genetic influence on methane emissions. Estimates of LSM for daily methane emissions among sire progeny groups showed significant differences ranging from 136 to 205 g/d (Fig 2). In contrast to the breed type effects, there were also significant differences between LSM for sire progeny group effects on methane emissions relative to the amount of feed consumed (Fig 2). Slightly different rankings of sires based on methane emissions per day in comparison to those based on per kg DMI are likely due to differences in feed intake among sire progeny groups. In some cases, the differences in methane emissions between sire progeny groups were even larger than the differences between the diets, indicating a substantial genetic influence of the host animal. The differences in LSM for methane emissions among sire progeny groups, as well as the similar ranking (r = 0.6) when methane emissions are expressed per day or per DMI, indicate that there is a direct genetic influence of the host on the rumen microbial methane production independent of the amount of feed consumed. Population genetic studies using beef cattle [10], dairy cattle [11] and sheep [12,13] lend supporting evidence for a genetic influence of the host on methane production. The advantage of the present study is that methane was measured using the considered “gold standard” measurement technique of respiration chambers and that the genetic and diet effects, as well as their interaction were estimated in a powerful experimental design under standardised conditions.

### Absence of interactions between host genetics and diets

There were no significant interactions between breed type (or sire) and diet effects in the present study. The absence of interactions indicates that the genetic ranking of sires would not change according to diet. This observation is of substantial importance for implementation of this approach within genetic improvement programmes, and should be confirmed in further independent studies. These results provide fundamental insight into the regulation of methane emissions, indicating that there is an additive genetic influence of the host on methane production by the archaea and that the genetic influence of the host on methane emissions does not change with the diet. The scaling effect of the diet on methane production could be adjusted for in genetic models. In contrast, if interactions between host genetics and diet are present, it would be necessary to use more complex selection strategies.

### Host genetics shape the microbiome

The host additive genetic influence on the microbiome was estimated based on differences in the archaea:bacteria ratio in rumen contents among sire progeny groups. Our earlier studies showed that the archaea:bacteria ratio in rumen contents from live animals can be used to predict methane emissions with a reasonable phenotypic correlation of 0.49 [14]. Other methods have also be investigated to predict methane emissions of animals e.g. the use of laser methane detector in sheep or beef cattle [18] and milk mid-infrared spectra in dairy cows, however, further discussion are beyond the scope of this study [19]. The ratio of archaea:bacteria in the rumen contents sample from each animal was more informative than the absolute amount of those microbes, most likely because the ratio is e.g. independent of dilution effects and differences in PCR amplification of 16S rRNA genes between samples. Comparison of sire progeny group estimates for the archaea:bacteria ratio (taken from live animals shortly after they left the respiration chambers) with those of methane emissions measured as g/day and kg/DMI showed similar ranking with correlations of r = 0.8 and 0.65, respectively. In addition, similar rank correlations (r = 0.72 and 0.67, respectively) with methane emissions were found for the archaea:bacteria ratios based on rumen contents samples taken in the abattoir even after a time lag between leaving the respiration chambers and slaughter of up to 15 days (Fig 2). Most of the deviations in ranks were due to two small progeny groups associated with the highest standard error. Therefore, the general consistency in ranking of sire progeny groups based on microbial and methane emission levels provides evidence that there is an additive genetic influence of the host on the rumen microbial community and their metabolic activity to produce methane. Thus, the archaea:bacteria ratio could be used as selection criteria for reduction of methane emissions. In particular, the rumen samples taken in the abattoir could be used to test a large cohort of sire progeny groups to accurately estimate their breeding values for methane emissions. Using the same experimental data, in a previous study we reported similar correlations between methane emissions per kg DMI and archaea:bacteria ratio in rumen contents samples taken in the abattoir as those taken from live animals [14], which opens up many opportunities to collect rumen microbial information as a basis for mitigating methane emissions and other traits such as feed conversion efficiency.

### Metagenomic gene abundance and methane emissions

**Fig 3:**
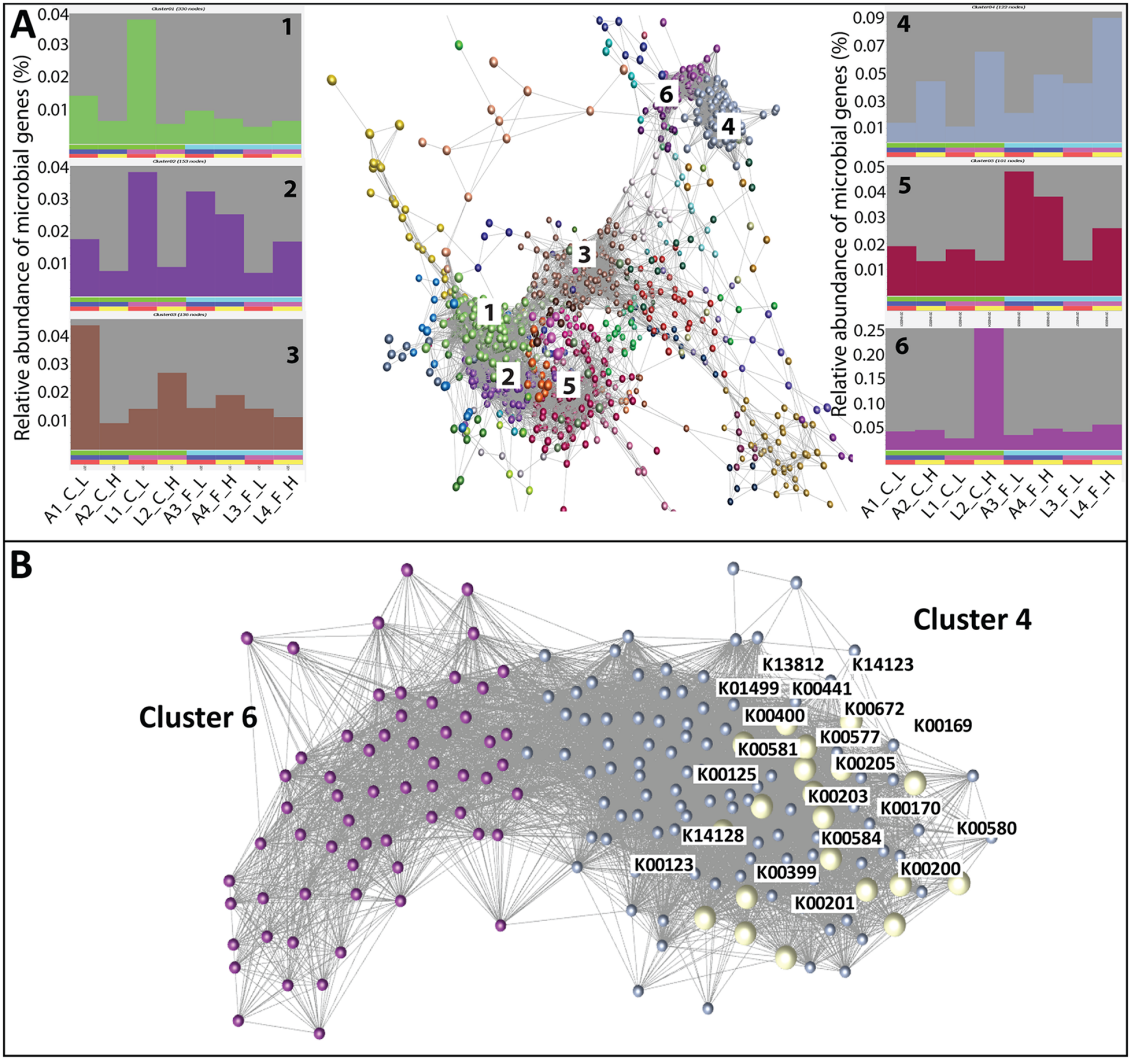
Functional clusters of microbial genes identified using network analysis. **(A)** Correlation analysis of microbial gene abundance was used to construct networks, where nodes represent microbial genes and edges the correlation in their abundance. Networks were clustered using the MCL algorithm and the profiles of clusters 1 to 6 are shown. Each chart represents the average abundance of genes in a cluster across the animals studied. Animals are ordered alternately being low (red bar beneath plot) and high methane emitter (yellow), whereby the first and last 4 bars represent animals offered concentrate (green) and forage (light blue) diets, respectively. See S2 Table for KEGG genes associated within each cluster. (B) Microbial gene networks of cluster 4 and 6 contained most of the microbial genes associated with methane metabolism; explicitly shown in yellow are the KEGG genes identified by the PLS analysis to be most closely associated with methane emissions (see Fig 4 and S3 Table).

To investigate the use of microbial gene abundance as an alternative selection criterion to mitigate methane emissions, the extreme animals for methane emissions within breed type and diet were selected and their rumen microbial genes determined using a metagenomic analysis. A previous study showed that the metagenomic data are highly informative, e.g. that higher abundance of the Proteobacteria *Succinovibrionaceae* was significantly associated with low emitting animals [20]. The high methane emission group had 88% higher emissions than the low group (S1 Fig). In the metagenomic study, 3970 KEGG genes (S2 Dataset) were identified in rumen contents samples taken in the abattoir, of which 1570 genes were used based on the relative abundance of more than 0.001% and the predictability within the univariate GLM analysis. The relative abundance of microbial genes is expected to be more informative than their absolute abundance because it is e.g. independent from dilution effects and the difference in amplifications of the genes between samples. Based on the relative abundance of these 1570 KEGG genes, we carried out a network analysis and found distinct functional clusters of gene networks (Fig 3A and S2 Table). In particular, cluster 4 and 6 formed a distinct group compared to all other clusters. Interestingly, these two clusters contained most genes known to be associated with methane metabolism (e.g. KEGG database pathway information). In contrast, in some other clusters, microbial genes directly or indirectly related to methane production occurred only sporadically e.g. cluster 13 (*phosphoenolpyruvate carboxylase*), cluster 21 (*acetate kinase*) and cluster 26 (*tetrahydromethanopterin S-methyltransferase subunit B)*. These clusters comprise only on a small number of genes and are well dispersed and distinct from cluster 4 and 6.

**Fig 4:**
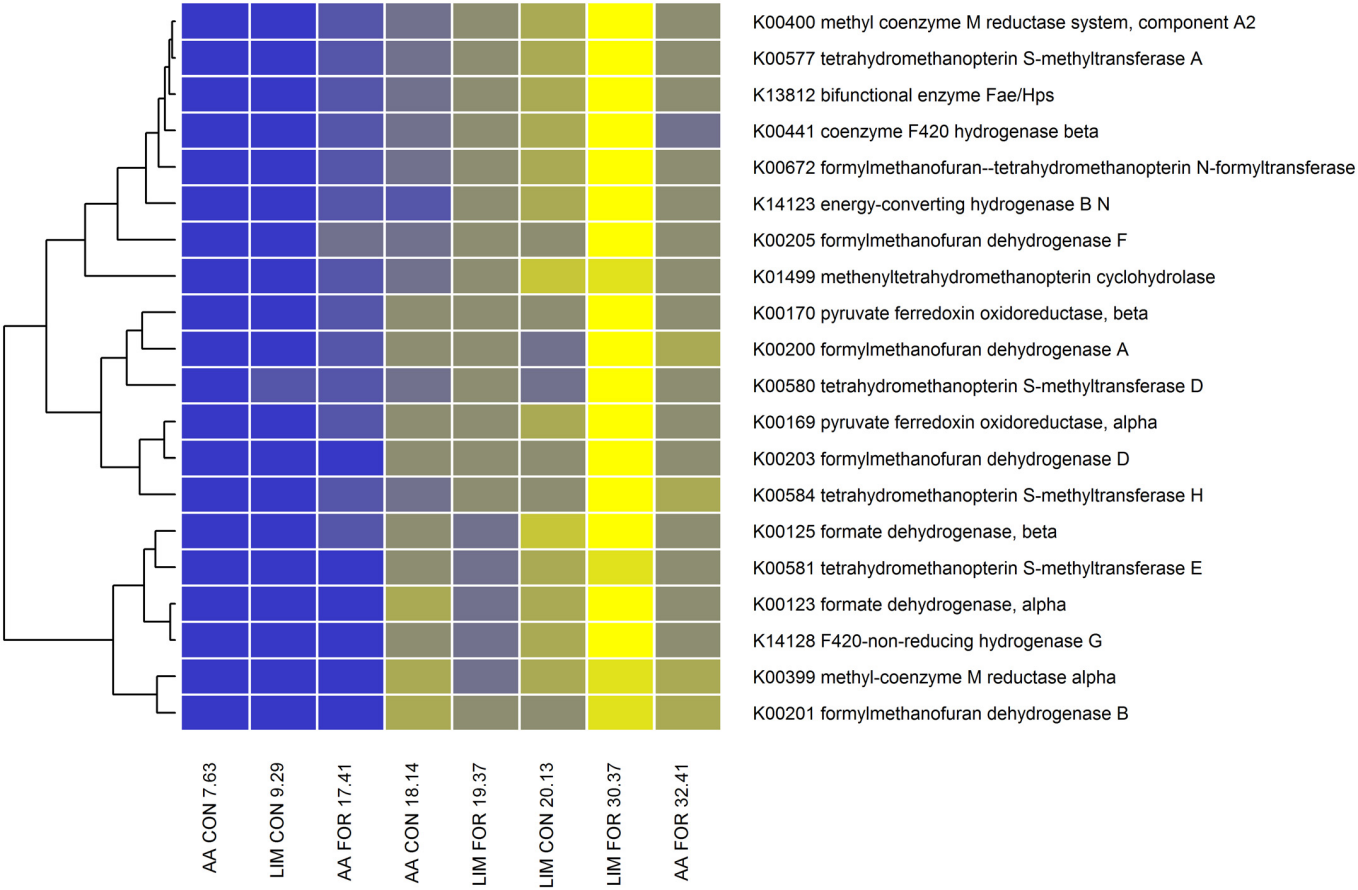
Heatmap of the relative abundance of microbial genes associated with methane emissions as identified in the partial least squares analysis. The relative abundance of microbial genes (blue = low to yellow = high) changed depending on methane emissions (g/kg DMI) for the animals selected for low and high methane emissions within breed type and diet. The labels on the horizontal axis indicate the crossbred breed type (AA = Aberdeen Angus sired, LIM = Limousin sired), diet (CON = concentrate based diet, FOR = forage based diet) and the amount of methane emissions (g/kg DMI).

To identify the importance of the different microbial genes to predict methane emissions we performed a partial least squares analysis, firstly on all microbial genes in cluster 4 and 6 and thereafter only on those genes directly stated in the literature or in the KEGG database to be involved in the methane metabolism pathway [20–24]. For further discussion of the microbial genes within metabolic pathways of methane metabolism see our previous study [20]. Using this information, the relative abundances of 20 microbial genes explained (including the diet effect) 81.7% of the variation in methane emissions (Fig 4 and S3 Table). The identified microbial genes are only in cluster 4 and interact closely with each other (Fig 3B). Excluding the diet effect from the model reduced the explained variation in methane emissions only slightly to 77.1%. However, inclusion of the diet effects in the prediction equation is recommended because then the influence of the microbial enzyme genes on methane emissions is estimated whilst taking diet effects into account. Based on a regression analysis of methane emission on the relative abundance of different microbial genes within diet we will later show that the slope of the regression lines are similar for the different diets and only shifted to a different level depending on the diet, as we would expect for a fixed effect. In general, the analysis suggests that methane emissions for the large cohort of animals necessary to obtain accurate genetic breeding values could be predicted accurately from the relative abundance of these 20 KEGG genes.

All analysed genomes of methanogenic archaea carry the *methyl-coenzyme M reductase alpha subunit* (*mcrA*) gene, which catalyses the last step in the methanogenesis [25,26]. Comparing the *mcrA* gene abundance between the low and high emission groups (170% increase) resulted in a highly significant difference (S2 Fig). An association between *mcrA* gene abundance and methane emissions has been reported in dairy cattle [27] and sheep (at the transcriptomic level) [28], whilst this gene is recommended for monitoring the process performance of anaerobic digesters [29]. Another identified archaeal gene was *formylmethanofuran dehydrogenase subunit B* (*fmdB*), which is also involved directly in methanogenesis and catalyses the reversible reduction of CO_2_ and methanofuran via N-carboxymethanofuran (carbamate) to N-formylmethanofuran, the first and second steps in the methanogenesis from CO_2_ [30,31]. For this gene, the high methane emissions group showed 173% greater relative abundance than the low group (S3 Fig). Within each of these genes, similar slopes of the regression lines for the diets provided were obtained which indicates that there were no interactions between microbial gene abundances and diet effects (S4 and S5 Figs). This is consistent with the absence of interactions between sire and diet effects described earlier. There was only a constant effect relating to the different diets, which can be considered as a fixed effect in the genetic evaluation model.

The study of [28] found a significant association between methane emissions and KEGG genes using data from metatranscriptomic, but not metagenomic, sequencing. In contrast, we obtained significant associations in data from metagenomic sequencing. This may be partly due to the higher statistical power of the present experiment, with a difference between selected high and low methane emitter groups of 11.8 g / kg DMI compared to 4.4 g / kg DMI in the study of [28].

### Metagenomic gene abundance and feed conversion efficiency

**Fig 5:**
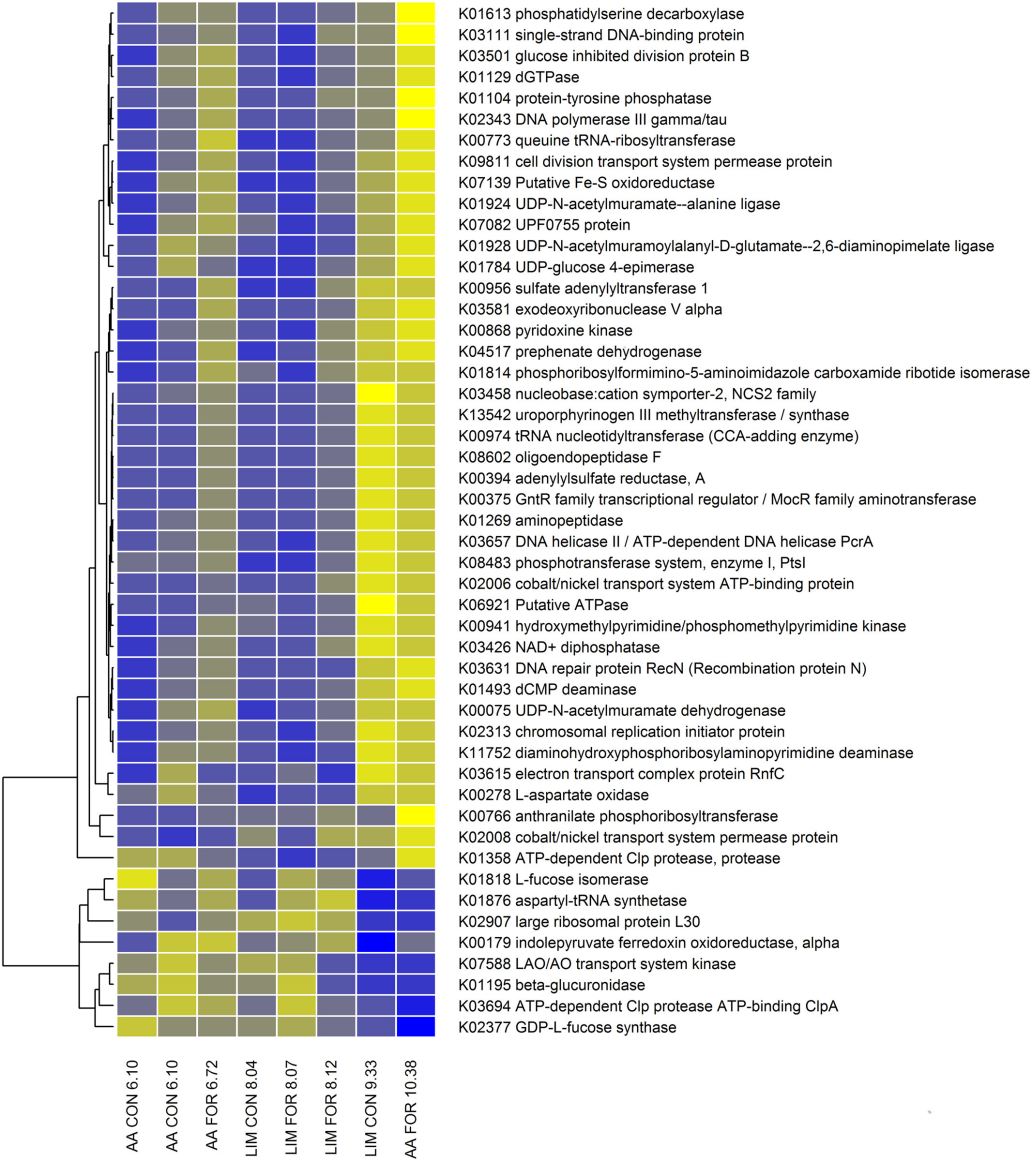
Heatmap of the relative abundance of microbial genes associated with feed conversion efficiency as identified in the partial least squares analysis. The relative abundance of microbial genes (blue = low to yellow = high) changed depending on feed conversion ratio (kg feed intake/kg growth) for the animals selected for low and high methane emissions within breed type and diet. The labels on the horizontal axis represent the breed type (AA = Aberdeen Angus sired, LIM = Limousin sired), diet (CON = concentrate based diet, FOR = forage based diet) and the feed conversion ratio (kg daily feed intake / kg daily growth).

The relative KEGG gene abundances need to be determined only once in a metagenomic study and can then be further used to investigate their relationship with other potential traits. In this study we also analysed association with feed conversion ratio and found that 49 genes were important to predict this trait and explained 88.3% of the variation (including breed type and diet effects) and 85.5% (excluding these effects) as illustrated in Fig 5 and summarised in S4 Table. Most of those genes were in clusters 2 and 5, indicating a close network of the genes associated with feed conversion efficiency (S3 Fig and S2 Table). However, these clusters were much more disperse and closer connected to other clusters than the clusters associated with methane. The reason is most likely that the animals were selected on the basis of extreme methane emissions, which provided more power to distinguish microbial gene networks associated with methane emissions than those related to feed conversion efficiency. The microbial genes associated with feed conversion efficiency encoded enzymes involved in host-microbe interactions (e.g. *GDP-L-fucose synthetase* (*TSTA3*), *L-fucose isomerase* (*FucI*)), the synthesis of amino acids and vitamins (e.g. *anthranilate phosphoribosyltransferase*, *uroporphyrinogen III methyltransferase*), degradation of amino acids and proteins (e.g. *aminopeptidase*), enzymes associated with genetic information processing (e.g. *aspartyl-tRNA synthetase*) and membrane processes (*cobalt/nickel transport system permease protein*). None of the 49 genes associated with feed conversion ratio were associated with methane emissions. Of particular interest is the abundance of *TSTA3* and *FucI*, which may reveal the importance of host-microbe cross talk in ruminants. These two genes related to feed conversion efficiency are involved in fucose metabolism. Fucose is a component of innate immunity glycoproteins (mucins) produced by the intestinal mucosa [32] and in saliva to help maintain the integrity of the mucosal barrier. ‘Fucose sensing’ has been identified as an important cross-talk between the intestinal microbiome and host tissues in studies with mice [33] and rabbits [34]. The degradation of mucins often requires enzymes from a range of bacteria, but some Bacteroides and Ruminococcus spp. are able to degrade mucins completely [35]. In particular, a cluster of bacterial genes involved in fucose uptake (FucP: L-*fucose permease*) and fucose utilisation (FucI: L-*fucose isomerase*; FucA: L-*fucose aldolase*; and FucK: L-*fucose kinase*) are controlled by a transcriptional repressor gene (FucR: L-*fucose operon activator*). FucR also controls bacterial signal production affecting host production of fucosylated glycans (i.e. mucins). This provides a mechanism to match bacterial demands for fucose with supply [35] and so affect the development of the microbiome [34].

Ross et al. [36] used the vector of counts of sequenced reads aligned to each contig in a database to create the metagenomic relationship matrix. We used an alternative approach of aligning the reads to identify the microbial genes first and then using the relative abundance of those genes to predict their influence on the trait of interest. The approach used in this study may have the advantage that the abundance of the microbial genes is highly related to the activity of the microbial ecosystem in the rumen.

### Possible mechanisms of host control of the gut microbiome

The mechanisms behind genetic influences of the host on the microbial community composition are expected to be based on many different biological factors. The pH of ruminal digesta is known to have a substantial effect on the microbial community structure and diversity in the rumen ecosystem. Saliva contains bicarbonate and tends to maintain rumen pH between 6 and 7 [37]. Adult cattle produce a substantial amount of saliva with an average of 150 L/day [38], though with substantial variation that is most likely influenced by host genetics as well as diet [39]. Furthermore, differences in bicarbonate secretion, short chain fatty acid absorption and passage rate of protons out of the rumen, all affect ruminal pH [37] and may be partly genetically determined. Variation in the physical structure and size of the rumen, as well as the intensity of contractions and rate of passage of digesta are all expected to have an influence on the rumen microbial community. Lower methane emissions were found in sheep with small rumens, most likely as a result of reduced digesta retention time [40]. Digesta retention time in ruminants has been shown to be heritable [41].

A recent review illustrates the highly complex interactions between microbiome and host [42], with the concept of microbiome-gut-brain axis interactions emerging. For example, the host’s central nervous system affects the gut microbiome through satiation signaling peptides affecting nutrient availability, hormones such as cortisol released by the hypothalamus-pituitary-adrenals axis during stress regulating gut contraction and integrity, and the immune system can be activated to alter gut flora—which links to ‘fucose sensing’ as discussed earlier based on our results.

### Microbial characteristics as selection criteria

We showed that there is an additive genetic effect of the host animal on the amount of methane produced by cattle via effects on the rumen microbial community. As a consequence, the characteristics of the rumen microbial community of each host (e.g. archaea:bacteria ratio) can be used as selection criteria to mitigate methane emissions. Even better prediction of methane emissions were obtained by using the relative abundance of microbial genes of each host. The relative abundance of rumen microbial genes can be related to any other trait associated with rumen function. In the present study, we demonstrated this for feed conversion efficiency, but there may also be associations between the relative abundance of microbial genes and animal health, meat quality, animal behaviour, milk composition, fatty acid composition, etc. Deep metagenomic sequencing remains relatively costly; therefore a functional metagenomic microarray to cost-effectively determine the relative abundance of rumen microbial genes would be a useful development. This would provide the opportunity to develop new selection strategies for these difficult to measure traits (e.g. methane emissions, feed conversion efficiency, animal health, and animal behaviour)—similar to the adoption of genome-wide selection in dairy breeding [43]. Using a reference population in which the traits of interest are measured, the prediction equations could be developed based on the relative abundance of rumen microbial genes and then used to predict e.g. methane emissions of other animals based only on the microbial composition in rumen contents samples without measuring the traits directly. Alternatively, the relative abundance of rumen microbial genes could be used directly for selection using e.g. relative weights equivalent to their effects on methane emissions. In addition, the recommended approach enables us to understand the biological significance of the specific genes in order to add further confidence for the application of the prediction equations. We may go further and hypothesize that selection of the host genetics based on the microbial gene abundances may be more efficient for improvement of feed conversion efficiency than using measured feed intake per unit of weight gained because the true conversion of feed may be more strongly related to the rumen microbial metabolism than to the measured feed conversion ratio, which are influenced by other factors (e.g. errors in measurements of feed intake and weight gain).

A further attraction of this approach is that the relative abundance of microbial genes can be based on rumen samples taken from either live or slaughtered animals so that efficient selection strategies can be developed using potential breeding animals as well as their slaughtered relatives. A potential adverse consequence of a successful selection of animals for reduced methane may be the accumulation of the substrate gas, H_2_, which is a product of fermentation by acetate and butyrate producing microorganisms, and that this accumulation would suppress fermentation rates in the rumen [44]. This result was founded mainly upon pure-culture studies in which H_2_ accumulation by a single H_2_-producing bacterial species resulted in thermodynamic inhibition of fermentation and growth [44–46]. Co-culture with a methanogen relieved this inhibition. As the main cellulolytic species are H_2_ producers, it was feared that preventing methane emissions would lead to H_2_ accumulation which would in turn slow fibre breakdown. The effects of H_2_ concentration are in fact much more complex [16]. Studies in gnotobiotic lambs lacking methanogens [47] and inhibiting methane emissions in goats and cattle using experimental halogenated compounds [48] suggested that growth was normal and other effects such as on feed intake were minor. The overall outcome of inhibiting methanogenesis seems to be fairly neutral, neither beneficial nor detrimental [49–51] although further research is necessary to clarify this issue.

More generally, the results may open up opportunities to use the relative abundance of microbial genes in the gastrointestinal tract of different species to predict their influence on traits e.g. health and behaviour. There is substantial evidence in humans that individuals harbour different microbial communities in their gut, with implications for host health in areas as diverse as obesity, cognitive function and allergy [52–54]. Experiments in rodents indicate a host-driven regulation of the gut microbiota that is genetically encoded [6–8]. Research in humans also indicates a host genetic influence on the gut microbiota using the abundance of microbial taxa, in particular the family of *Christensenellaceae*, which formed a co-occurrence network with other bacteria and with methanogenic archaea and impacts metabolism [9]. Here, we show that the use of the abundance of the microbial genes is much more closely associated with metabolism than the abundance of the microbial community (for which the archaea:bacteria ratio was the best predictor [14]) and therefore a much better criterion to predict the host genetic influence on those traits. In addition, specific microbial genes, their networks and pathways can be used to better understand the association between host genetics and microbial activity related to the trait of interest. This could provide opportunities for personalized medicine considering the genetic link between host and microbiome and its activity, e.g. for treatment of inflammatory bowel disease in humans, which showed strong host-microbe interactions [55].

## Materials and Methods

### Ethics statement

This study was conducted at the Beef and Sheep Research Centre of Scotland’s Rural College (6 miles south of Edinburgh, UK). The experiment was approved by the Animal Experiment Committee of SRUC and was conducted in accordance with the requirements of the UK Animals (Scientific Procedures) Act 1986.

### Sample collection and experimental design

The data were obtained from a 2 × 2 factorial design experiment of breed types and diets using 72 steers from a two-breed rotational cross between AA and LIM. Equal numbers of experimental animals were sired by purebred AA and LIM. Depending on the purebred sire used, the expected additive genetic contributions were 2/3 and 1/3 from each of the two breeds. Progeny groups were from 5 AA and 4 LIM sires. The average number (range) of progenies per sire were 7 (2 to 12) and 9 (6 to 14) for AA and LIM, respectively. The animals were offered two complete diets *ad libitum* consisting (g/kg DM) of either 480 forage to 520 concentrate or 75 forage to 925 concentrate; these are subsequently described as forage and concentrate diets, respectively. The detailed diet composition has been published by [56].

### Animals, husbandry and measurements

The growing-finishing beef cattle were bred, raised and performance tested at the Beef and Sheep Research Centre of SRUC. Before artificial insemination (AI), the dams of the experimental animals were housed outdoors at grass. All dams were synchronised for AI using Progesterone (Eazi-Breed CIDR cattle insert, Zoetis UK Ltd., UK), Estrumate, PMSG and Prostaglandin, (Intervet UK Ltd., UK). AI took place from June through to August 2009. All cows were transferred indoors at the beginning of November 2009 where they remained in group-pens until calving which took place from March through to May 2010. At late spring time, cows with the experimental calves at foot were transferred outdoors and kept on grass until mid-November. Cows and calves were transferred to indoor group-pens and after few days calves were weaned. Weaned calves were transferred to group-pens at the facility until the experiment commenced. All dams were routinely vaccinated for leptospirosis (Leptavoid H, Intervet UK Ltd., UK), bovine viral diarrhoea (BVD) (Bovilis, Intervet UK Ltd., UK) and rotavirus (Rotavec Corona, Intervet UK Ltd., UK) and treated for nematodes, lice and mites (Dectomax, Zoetis UK Ltd., UK). All calves were routinely vaccinated for infectious bovine rhinotracheitis, BVD, Bovine Parainfluenza 3 virus and Bovine Respiratory Syncytial Virus (Rispoval 4, Zoetis UK Ltd, UK) and treated for nematodes, lice and mites (Dectomax, Zoetis UK Ltd., UK). Due to EU legislation the application of hormones enhancing growth is prohibited and antibiotics and drugs were only administered in exceptional cases and those animals were excluded from the trial. All mothers with calves were offered the same diet each day.

No twin calves were used in the trial so that each experimental animal had its own specific maternal effect influencing the microbiota. Prior to the start of the trial, animals were adapted to the experimental diets over a 5 week period. During this period, the animals acclimatised to the group-housed environment and were trained to use the electronic feeders (HOKO, Insentec, Marknesse, The Netherlands).

During the performance test period of 56 days, the animals were group-housed in two pens of 36 each, balanced for breed type. Within each pen, half of the animals had access to one of the two diets, again balanced for breed type. In addition, treatments were balanced for age at start of test and body weight. All animals were bedded on wood fines to ensure that there was no consumption of bedding. Using electronic feeders, daily feed intake was recorded and daily dry matter intake (DMI, kg/day) calculated using analysed dry matter content of duplicated samples of each diet component taken twice weekly. Body weight of each animal was measured weekly and average daily gain (ADG) was obtained by fitting a linear regression of body weight on test date. Feed conversion ratio (FCR) was calculated as average DMI per day divided by ADG.

One week before entering the respiration chambers, the animals were housed individually in training pens, identical in size and shape to the pens inside the chambers, to allow them to adapt to being housed individually. Methane emissions were individually measured for 48h within 6 respiration chambers. The animals were allocated to the respiration chambers in a randomised block design with 3 replicates. Data from 4 animals could not be considered due to health issues and an air leak in one of the respiration chamber. The method of measurement in the respiration chambers is described in detail by [56].

### Genomic analysis

Rumen samples were obtained from the animals when they were alive (n = 50) and after slaughter (n = 68). Rumen samples were taken from live animals within 2 hours of leaving the respiration chambers. Approximately 50 mL rumen contents were taken by inserting a stomach tube (16 × 2700 mm Equivet Stomach Tube, JørgenKruuse A/S, Langeskov, Denmark) nasally and aspirating manually. Between 3 to 17 days after leaving the respiration chamber the animals were slaughtered in a commercial abattoir where two rumen fluid samples (approximately 50 mL) were taken immediately after the rumen was opened to be drained. The slaughter process results in well mixed samples of rumen contents. DNA was extracted from the rumen samples and subjected to qPCR for the 16S rRNA genes as described in [14] to determine the abundance of archaea and bacteria and their ratio.

Eight extreme animals (4 high and 4 low) for methane emissions, balanced for breed type and diet, were used in a metagenomic study, in which deep sequencing was applied. Illumina TruSeq libraries were prepared from genomic DNA and sequenced on an Illumina HiSeq 2500 instrument by Edinburgh Genomics. Paired-end reads (2 × 100 bp) were generated, resulting in between 8.6 and 14.5 GB per sample (between 43.4 and 72.7 million paired reads). The genomic reads were aligned to the KEGG genes database. Parameters were adjusted such that all hits were reported that were equal in quality to the best hit for each genomic read. The read and best hits have to be more than 90% identical and have to be belonging to a single KEGG orthologue group to be kept in the data. If the best hits are spread over more than one KEGG orthologue group, the read were disregarded. Read counts for KEGG orthologues were summed and normalised to the total number of hits.

### Statistical analysis

For the analysis of methane emissions and qPCR determined microbial KINGDOM (Archaea and Bacteria), least squares means (LSM) were estimated using a general linear model analysis (GLM, Version 9.1 for Windows, SAS Institute Inc., Cary, NC, USA), including the effects of breed type (or sire within breed type), diet, respiration chamber and randomised block. Using a sire model with each progeny originating from a different mother, maternal effects are expected to be included in the residual effects and therefore did not bias the estimated LSM of sires. In a network analysis using BioLayout *Express*^3D^ [57] we identified the distinct functional clusters of microbial genes. These networks consist of nodes representing microbial genes and the connecting edges determining the functional linkages between these genes. Preliminary GLM analysis was carried out to estimate the influence of the KEGG genes on methane emissions and feed conversion efficiency by fitting the significant effects (diet for methane emissions, diet and breed type for feed conversion efficiency) as well as the relative abundance of one KEGG gene each time. The residuals of each model were normal distributed.

We used partial least squares analysis (PLS, Version 9.1 for Windows, SAS Institute Inc., Cary, NC, USA) to identify the most important of genes association with methane and feed conversion efficiency. The PLS analysis accounts for multiple testing and the correlation between microbial genes. In addition to microbial genes, the model included the diet effect (for methane emissions) and additionally the breed type effect (for feed conversion ratio). The model selection were based on the variable importance for projection (VIP) criterion [58], whereby microbial genes with a VIP < 0.8 contribute little to the prediction. In the PLS analysis to predict methane emissions, microbial genes of the two gene network clusters (4 and 6) which included most of the genes associated with methane metabolism were used, whereas for feed conversion ratio we used all KEGG genes that had a P-value <0.1 in the GLM analysis. Different strategies for the two analysed traits were applied because the animals were selected based on maximum differences in magnitude of methane emissions, so that the network analysis showed most discrimination for methane emissions.

## Supporting Information

S1 Fig Difference in least squares means of methane emissions in groups of low and high emitting animals. Methane emissions were measured in g/kg feed dry matter intake (DMI) in respiration chambers and the estimates were adjusted for diet effects.
(TIFF)

S2 Fig Comparison of the relative abundance of methyl-coenzyme M reductase alpha subunit (*mcrA*) gene for low and high methane emitting animals. The metagenomic analysis was based on samples of rumen contents taken *post mortem*. Least squares means of relative abundance of *mcrA* were adjusted for diet effects.
(TIFF)

S3 Fig Comparison of the relative abundance of formylmethanofuran dehydrogenase subunit B (*fmdB*) gene for low and high methane emitting animals. The metagenomic analysis was based on samples of rumen contents taken *post mortem*. Least squares means of relative abundance of *fmdB* were adjusted for diet effects.
(TIFF)

S4 Fig Regression of methane emissions on the relative abundance of methyl-coenzyme M reductase alpha subunit (*mcrA*) within diet. The metagenomic analysis was based on samples of rumen contents taken *post mortem*. Methane emissions were measured in g/kg feed dry matter intake (DMI) using respiration chambers.
(TIFF)

S5 Fig Regression of methane emissions on the relative abundance of formylmethanofuran dehydrogenase subunit B (*fmdB*) within diet. The metagenomic analysis was based on samples of rumen contents taken *post mortem*. Methane emissions were measured in g/kg feed dry matter intake (DMI) using respiration chambers.
(TIF)

S1 Table Means and variability of methane emissions and archaea:bacteria ratios for Aberdeen Angus- or Limousin-sired cattle offered either forage- or concentrate-based diets. (PDF)

S2 Table Functional microbial gene network clusters. In a network analysis using BioLayout Express^3D^ distinct functional clusters of microbial genes were identified based their relative abundances in the rumen samples from animals. The functional clusters 4 and 6 contained most of the microbial genes associated with methane metabolism, whereas clusters 2 and 5 included most of the genes related to feed conversion efficiency.
(PDF)

S3 Table Microbial genes associated with methane emissions. Partial least squares (PLS) estimates of effects of microbial genes characterised by the Kyoto Encyclopedia of Genes and Genomes (KEGG) database in an analysis where the PLS factors explained 97.0% of the variation of model effects and 81.7% of the variation in methane emissions (g/kg dry matter intake).
(PDF)

S4 Table Microbial genes associated with feed conversion efficiency. Partial least squares (PLS) estimates of effects of microbial genes characterised by the Kyoto Encyclopedia of Genes and Genomes (KEGG) database in an analysis where the PLS factors explained 80.6% of the variation of model effects and 88.3% of the variation in feed conversion ratio (kg feed intake/kg growth).
(PDF)

S1 Dataset Data used for prediction of host genetic effects. (XLSX)

S2 Dataset Metagenomic data for the analysis of the associations between relative abundances of microbial genes with methane emissions and feed conversion efficiency. (XLSX)
