# Supplementary figures and images for: Bovine Host Genetic Variation Influences Rumen Microbial Methane Production with Best Selection Criterion for Low Methane Emitting and Efficiently Feed Converting Hosts Based on Metagenomic Gene Abundance
Source: PLoS Genet. 2016 Feb 18;12(2):e1005846. doi: 10.1371/journal.pgen.1005846 (PMC4758630; doi:10.1371/journal.pgen.1005846)

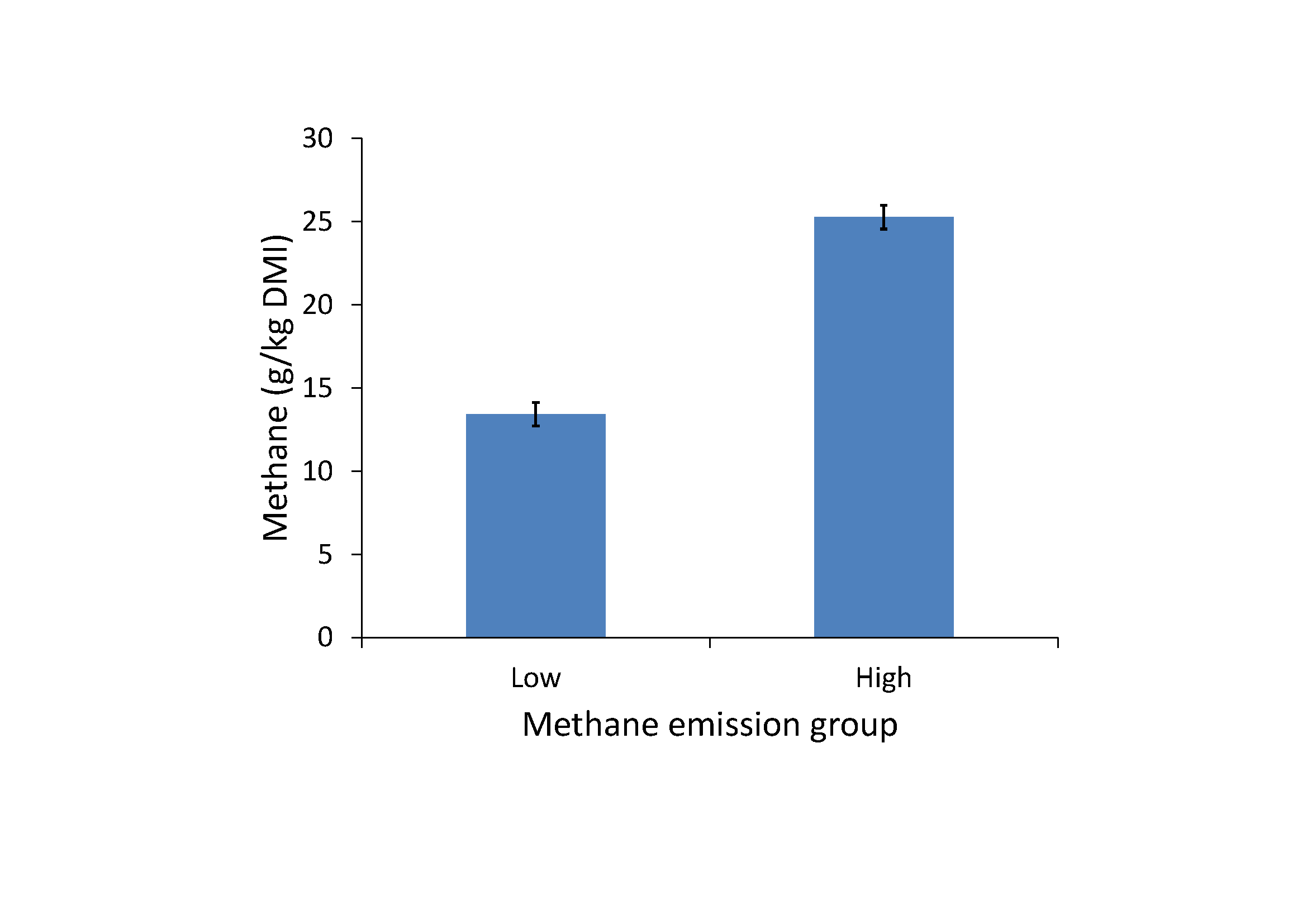

Supplement: S1 Fig — Methane emissions were measured in g/kg feed dry matter intake (DMI) in respiration chambers and the estimates were adjusted for diet effects. (TIFF) [file pgen.1005846.s001.tiff]

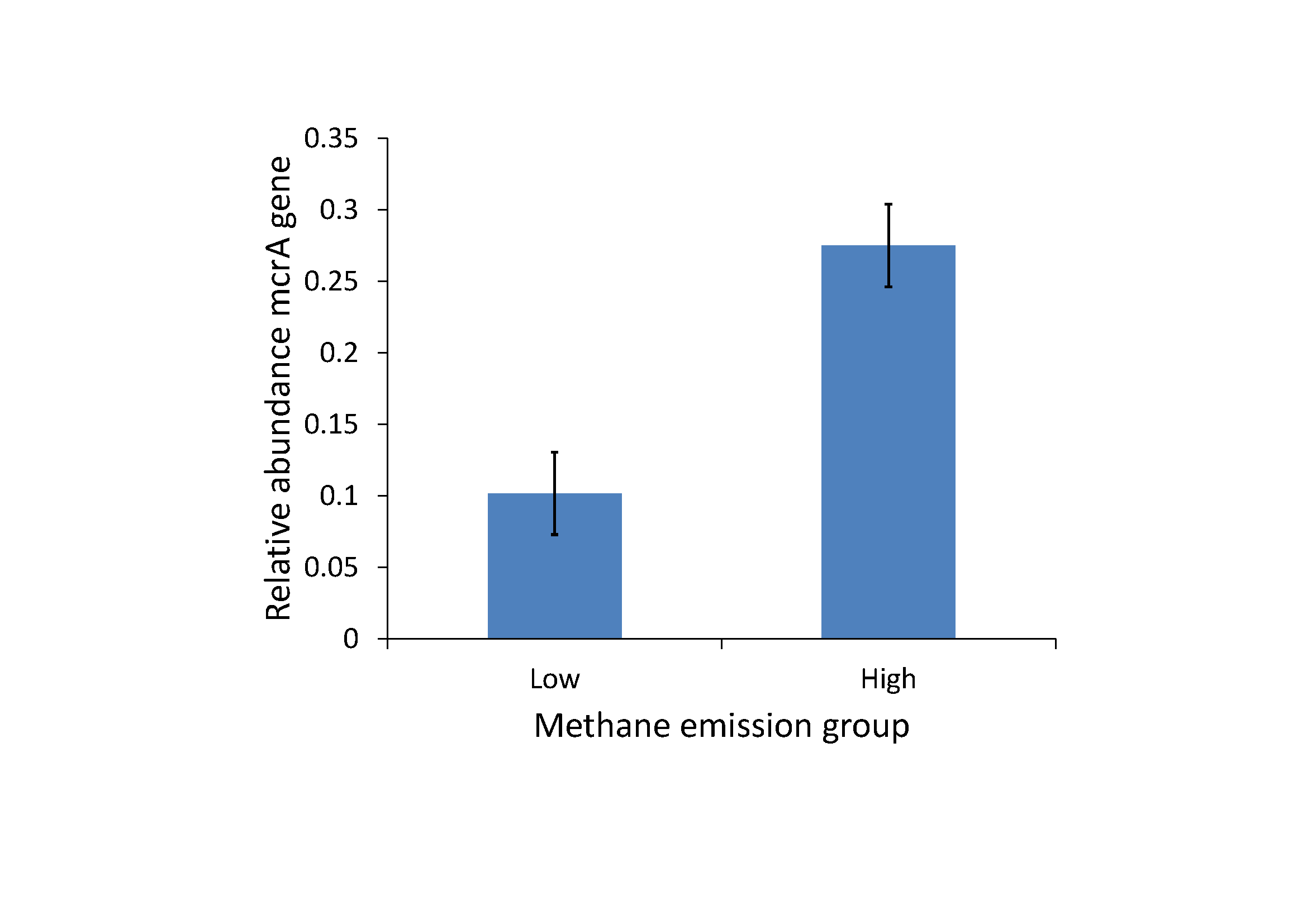

Supplement: S2 Fig — The metagenomic analysis was based on samples of rumen contents taken post mortem. Least squares means of relative abundance of mcrA were adjusted for diet effects. (TIFF) [file pgen.1005846.s002.tiff]

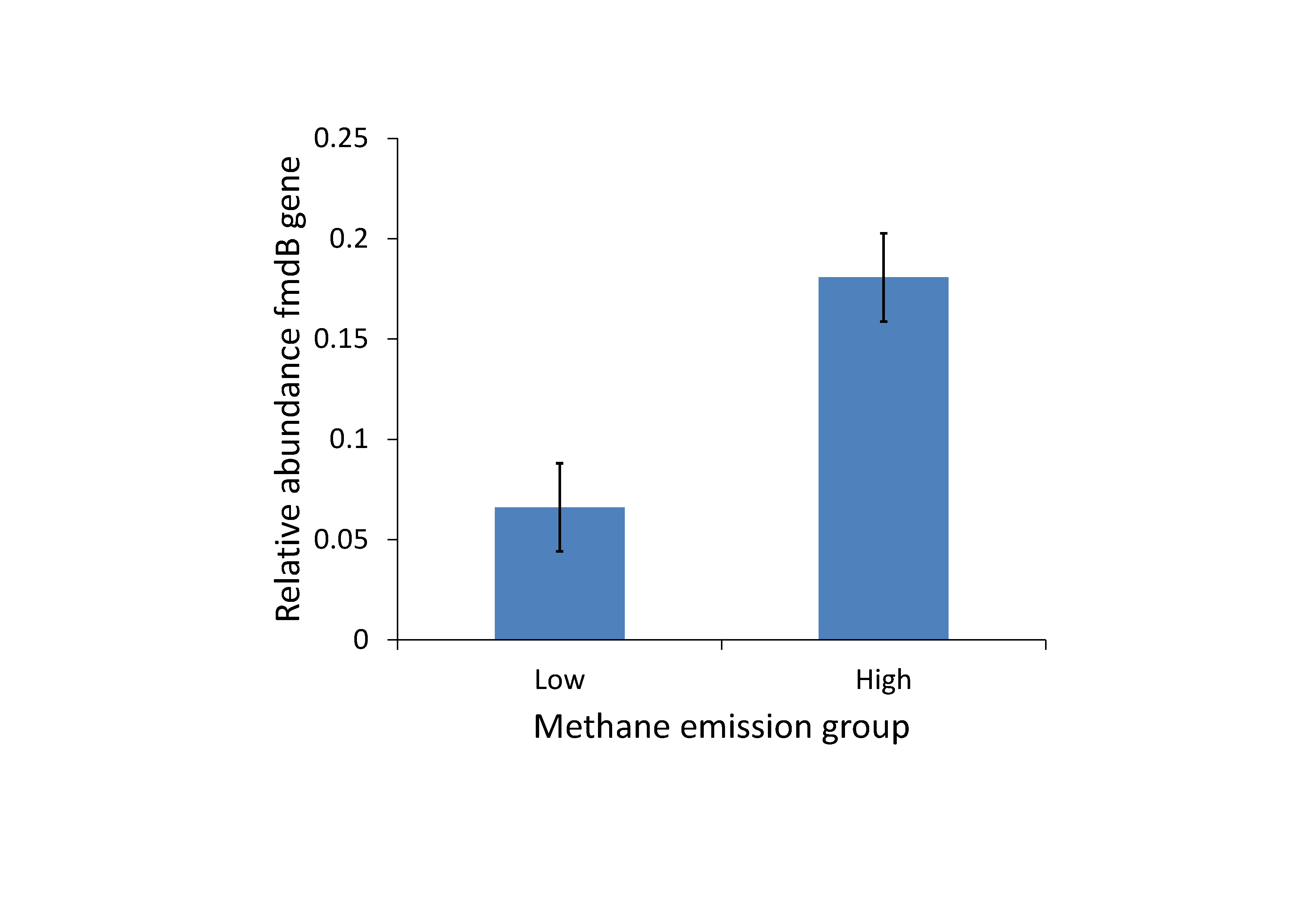

Supplement: S3 Fig — The metagenomic analysis was based on samples of rumen contents taken post mortem. Least squares means of relative abundance of fmdB were adjusted for diet effects. (TIFF) [file pgen.1005846.s003.tiff]

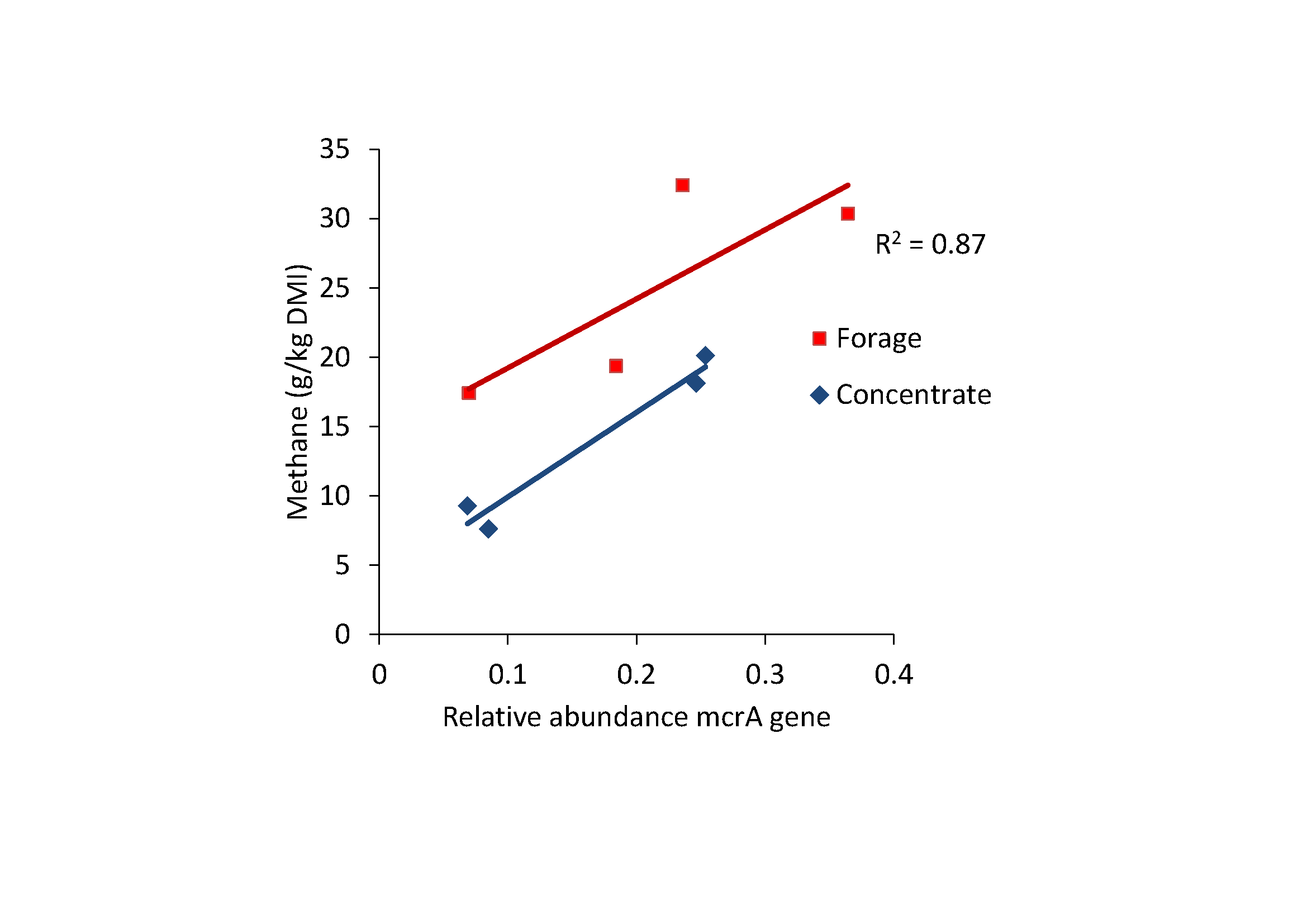

Supplement: S4 Fig — The metagenomic analysis was based on samples of rumen contents taken post mortem. Methane emissions were measured in g/kg feed dry matter intake (DMI) using respiration chambers. (TIFF) [file pgen.1005846.s004.tiff]

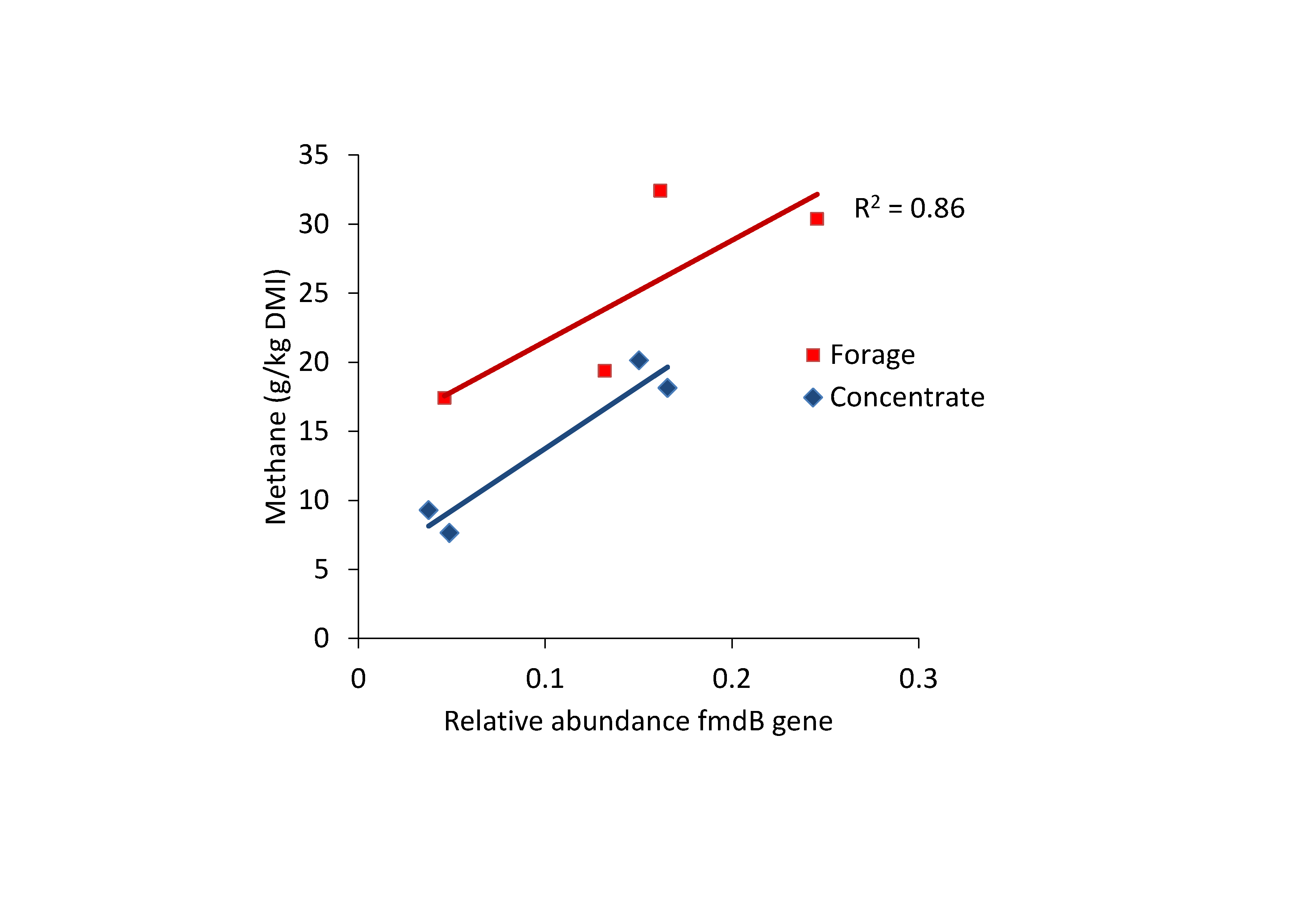

Supplement: S5 Fig — The metagenomic analysis was based on samples of rumen contents taken post mortem. Methane emissions were measured in g/kg feed dry matter intake (DMI) using respiration chambers. (TIF) [file pgen.1005846.s005.tif]
