# Supplementary material for: Bovine Host Genetic Variation Influences Rumen Microbial Methane Production with Best Selection Criterion for Low Methane Emitting and Efficiently Feed Converting Hosts Based on Metagenomic Gene Abundance
Source: PLoS Genet. 2016 Feb 18;12(2):e1005846. doi: 10.1371/journal.pgen.1005846 (PMC4758630; doi:10.1371/journal.pgen.1005846)
Supplement: S1 Table — (PDF) [file pgen.1005846.s006.pdf]

Table S1. Means and variability of methane emissions and archaea:bacteria ratios for Aberdeen Angus- or Limousin-sired cattle offered either forage- or concentrate-based diets.

		Forage based diet				Concentrate based diet			
**Trait**	**Breed type**	**No**	**Mean**	**CV (%)**	**Range**	**No**	**Mean**	**CV (%)**	**Range**
Methane, g/day	Aberdeen Angus sired	17	218.2	17.6	172 - 333	18	149.6	25.1	78 - 233
	Limousin sired	17	192.4	14.4	152 - 266	16	136.1	31.5	86 - 216
Methane, g/kg DMI	Aberdeen Angus sired	17	21.38	19.4	15.90 - 31.41	18	13.42	23.8	7.63 - 18.14
	Limousin sired	17	22.03	17.7	14.43 - 30.37	16	13.93	28.8	9.29 - 22.78
Archaea:Bacteria ratio	Aberdeen Angus sired	13	7.73	50.1	3.05 – 17.11	14	3.33	64.5	0.70 - 8.47
in live animals	Limousin sired	12	5.87	39.0	2.13 – 9.38	11	2.64	65.1	0.96 - 6.73
Archaea:Bacteria ratio	Aberdeen Angus sired	17	6.37	35.4	1.47 – 10.95	17	2.75	50.2	0.85 - 5.77
in slaughtered animals	Limousin sired	17	6.87	42.6	2.23 – 14.0	16	2.97	39.3	1.38 - 4.91

DMI = dry matter intake; CV = Coefficient of variation.
