# Supplementary material for: Bovine Host Genetic Variation Influences Rumen Microbial Methane Production with Best Selection Criterion for Low Methane Emitting and Efficiently Feed Converting Hosts Based on Metagenomic Gene Abundance
Source: PLoS Genet. 2016 Feb 18;12(2):e1005846. doi: 10.1371/journal.pgen.1005846 (PMC4758630; doi:10.1371/journal.pgen.1005846)
Supplement: S2 Table — In a network analysis using BioLayout Express3D distinct functional clusters of microbial genes were identified based their relative abundances in the rumen samples from animals. The functional clusters 4 and 6 contained most of the microbial genes associated with methane metabolism, whereas clusters 2 and 5 included most of the genes related to feed conversion efficiency. (PDF) [file pgen.1005846.s007.pdf]

Table S2. Functional microbial gene network clusters. In a network analysis using BioLayout Express^{3D} distinct functional clusters of microbial genes were identified based their relative abundances in the rumen samples from animals. The functional clusters 4 and 6 contained most of the microbial genes associated with methane metabolism, whereas clusters 2 and 5 included most of the genes related to feed conversion efficiency.

**KEGG-ID**	**Description**	**Cluster**
K00005	glycerol dehydrogenase [EC:1.1.1.6]	1
K00016	L-lactate dehydrogenase [EC:1.1.1.27]	1
K00027	malate dehydrogenase (oxaloacetate-decarboxylating) [EC:1.1.1.38]	1
K00048	lactaldehyde reductase [EC:1.1.1.77]	1
K00050	hydroxypyruvate reductase [EC:1.1.1.81]	1
K00096	glycerol-1-phosphate dehydrogenase [NAD(P)] [EC:1.1.1.261]	1
K00104	glycolate oxidase [EC:1.1.3.15]	1
K00111	glycerol-3-phosphate dehydrogenase [EC:1.1.5.3]	1
K00135	succinate-semialdehyde dehydrogenase (NADP+) [EC:1.2.1.16]	1
K00248	butyryl-CoA dehydrogenase [EC:1.3.99.2]	1
K00260	glutamate dehydrogenase [EC:1.4.1.2]	1
K00284	glutamate synthase (ferredoxin) [EC:1.4.7.1]	1
K00366	ferredoxin-nitrite reductase [EC:1.7.7.1]	1
K00370	nitrate reductase 1, alpha subunit [EC:1.7.99.4]	1
K00378	hydroxylamine reductase [EC:1.7.-.-]	1
K00384	thioredoxin reductase (NADPH) [EC:1.8.1.9]	1
K00385	anaerobic sulfite reductase subunit C [EC:1.8.1.-]	1
K00533	ferredoxin hydrogenase large subunit [EC:1.12.7.2]	1
K00547	homocysteine S-methyltransferase [EC:2.1.1.10]	1
K00558	DNA (cytosine-5-)-methyltransferase [EC:2.1.1.37]	1
K00595	precorrin-6Y C5,15-methyltransferase / precorrin-8W decarboxylase [EC:2.1.1.132 1.-.-.-]	1
K00603	glutamate formiminotransferase [EC:2.1.2.5]	1
K00616	transaldolase [EC:2.2.1.2]	1
K00681	gamma-glutamyltranspeptidase [EC:2.3.2.2]	1
K00690	sucrose phosphorylase [EC:2.4.1.7]	1
K00763	nicotinate phosphoribosyltransferase [EC:2.4.2.11]	1
K00784	ribonuclease Z [EC:3.1.26.11]	1
K00788	thiamine-phosphate pyrophosphorylase [EC:2.5.1.3]	1
K00797	spermidine synthase [EC:2.5.1.16]	1
K00805	heptaprenyl diphosphate synthase [EC:2.5.1.30]	1
K00821	acetylornithine/N-succinyldiaminopimelate aminotransferase [EC:2.6.1.11 2.6.1.17]	1
K00850	6-phosphofructokinase [EC:2.7.1.11]	1
K00878	hydroxyethylthiazole kinase [EC:2.7.1.50]	1
K00882	1-phosphofructokinase [EC:2.7.1.56]	1
K00891	shikimate kinase [EC:2.7.1.71]	1
K00919	4-diphosphocytidyl-2-C-methyl-D-erythritol kinase [EC:2.7.1.148]	1
K00926	carbamate kinase [EC:2.7.2.2]	1
K00937	polyphosphate kinase [EC:2.7.4.1]	1
K00954	pantetheine-phosphate adenylyltransferase [EC:2.7.7.3]	1

Continued on next page

Table S2 – Continued from previous page

K00965	UDPglucose--hexose-1-phosphate uridylyltransferase [EC:2.7.7.12]	1
K00975	glucose-1-phosphate adenylyltransferase [EC:2.7.7.27]	1
K00996	undecaprenyl-phosphate galactose phosphotransferase [EC:2.7.8.6]	1
K01008	selenide, water dikinase [EC:2.7.9.3]	1
K01039	glutaconate CoA-transferase, subunit A [EC:2.8.3.12]	1
K01040	glutaconate CoA-transferase, subunit B [EC:2.8.3.12]	1
K01077	alkaline phosphatase [EC:3.1.3.1]	1
K01091	phosphoglycolate phosphatase [EC:3.1.3.18]	1
K01126	glycerophosphoryl diester phosphodiesterase [EC:3.1.4.46]	1
K01139	guanosine-3 ,5 -bis(diphosphate) 3 -pyrophosphohydrolase [EC:3.1.7.2]	1
K01151	deoxyribonuclease IV [EC:3.1.21.2]	1
K01153	type I restriction enzyme, R subunit [EC:3.1.21.3]	1
K01155	type II restriction enzyme [EC:3.1.21.4]	1
K01163	hypothetical protein	1
K01187	alpha-glucosidase [EC:3.2.1.20]	1
K01193	beta-fructofuranosidase [EC:3.2.1.26]	1
K01246	DNA-3-methyladenine glycosylase I [EC:3.2.2.20]	1
K01258	tripeptide aminopeptidase [EC:3.4.11.4]	1
K01295	glutamate carboxypeptidase [EC:3.4.17.11]	1
K01304	pyroglutamyl-peptidase [EC:3.4.19.3]	1
K01421	putative membrane protein	1
K01436	aminoacylase [EC:3.5.1.14]	1
K01442	choloylglycine hydrolase [EC:3.5.1.24]	1
K01467	beta-lactamase [EC:3.5.2.6]	1
K01468	imidazolonepropionase [EC:3.5.2.7]	1
K01480	agmatinase [EC:3.5.3.11]	1
K01488	adenosine deaminase [EC:3.5.4.4]	1
K01514	exopolyphosphatase [EC:3.6.1.11]	1
K01515	ADP-ribose pyrophosphatase [EC:3.6.1.13]	1
K01531	Mg2+-importing ATPase [EC:3.6.3.2]	1
K01533	Cu2+-exporting ATPase [EC:3.6.3.4]	1
K01534	Cd2+/Zn2+-exporting ATPase [EC:3.6.3.3 3.6.3.5]	1
K01571	oxaloacetate decarboxylase, alpha subunit [EC:4.1.1.3]	1
K01581	ornithine decarboxylase [EC:4.1.1.17]	1
K01615	glutaconyl-CoA decarboxylase [EC:4.1.1.70]	1
K01626	3-deoxy-7-phosphoheptulonate synthase [EC:2.5.1.54]	1
K01643	citrate lyase subunit alpha / citrate CoA-transferase [EC:4.1.3.6 2.8.3.10]	1
K01644	citrate lyase subunit beta / citryl-CoA lyase [EC:4.1.3.6 4.1.3.34]	1
K01658	anthranilate synthase component II [EC:4.1.3.27]	1
K01682	aconitate hydratase 2 [EC:4.2.1.3]	1
K01706	glucarate dehydratase [EC:4.2.1.40]	1
K01708	galactarate dehydratase [EC:4.2.1.42]	1
K01735	3-dehydroquinate synthase [EC:4.2.3.4]	1
K01751	diaminopropionate ammonia-lyase [EC:4.3.1.15]	1
K01754	threonine dehydratase [EC:4.3.1.19]	1
K01759	lactoylglutathione lyase [EC:4.4.1.5]	1

Continued on next page

Table S2 – Continued from previous page

K01817	phosphoribosylanthranilate isomerase [EC:5.3.1.24]	1
K01839	phosphopentomutase [EC:5.4.2.7]	1
K01878	glycyl-tRNA synthetase alpha chain [EC:6.1.1.14]	1
K01879	glycyl-tRNA synthetase beta chain [EC:6.1.1.14]	1
K01910	[citrate (pro-3S)-lyase] ligase [EC:6.2.1.22]	1
K01950	NAD+ synthase (glutamine-hydrolysing) [EC:6.3.5.1]	1
K01961	acetyl-CoA carboxylase, biotin carboxylase subunit [EC:6.4.1.2 6.3.4.14]	1
K01978	M1 RNA	1
K01989	putative ABC transport system substrate-binding protein	1
K01992	ABC-2 type transport system permease protein	1
K01995	branched-chain amino acid transport system ATP-binding protein	1
K01997	branched-chain amino acid transport system permease protein	1
K01998	branched-chain amino acid transport system permease protein	1
K02011	iron(III) transport system permease protein	1
K02012	iron(III) transport system substrate-binding protein	1
K02013	iron complex transport system ATP-binding protein [EC:3.6.3.34]	1
K02029	polar amino acid transport system permease protein	1
K02036	phosphate transport system ATP-binding protein [EC:3.6.3.27]	1
K02037	phosphate transport system permease protein	1
K02038	phosphate transport system permease protein	1
K02040	phosphate transport system substrate-binding protein	1
K02114	F-type H+-transporting ATPase subunit epsilon [EC:3.6.3.14]	1
K02227	adenosylcobinamide-phosphate synthase CobD [EC:6.3.1.10]	1
K02231	adenosylcobinamide kinase / adenosylcobinamide-phosphate guanylyltransferase [EC:2.7.1.156 2.7.7.62]	1
K02233	adenosylcobinamide-GDP ribazoletransferase [EC:2.7.8.26]	1
K02315	DNA replication protein DnaC	1
K02334	DNA polymerase bacteriophage-type [EC:2.7.7.7]	1
K02371	enoyl-[acyl carrier protein] reductase II [EC:1.3.1.-]	1
K02392	flagellar basal-body rod protein FlgG	1
K02400	flagellar biosynthesis protein FlhA	1
K02406	flagellin	1
K02407	flagellar hook-associated protein 2	1
K02410	flagellar motor switch protein FliG	1
K02412	flagellum-specific ATP synthase [EC:3.6.3.14]	1
K02435	aspartyl-tRNA(Asn)/glutamyl-tRNA (Gln) amidotransferase subunit C [EC:6.3.5.6 6.3.5.7]	1
K02438	glycogen operon protein GlgX [EC:3.2.1.-]	1
K02446	fructose-1,6-bisphosphatase II [EC:3.1.3.11]	1
K02471	putative ATP-binding cassette transporter	1
K02495	oxygen-independent coproporphyrinogen III oxidase [EC:1.3.99.22]	1
K02499	tetrapyrrole methylase family protein / MazG family protein	1
K02502	ATP phosphoribosyltransferase regulatory subunit	1

Continued on next page

K02507	protein transport protein HofQ	1
K02529	LacI family transcriptional regulator	1
K02566	NagD protein	1
K02647	carbohydrate diacid regulator	1
K02777	PTS system, glucose-specific IIA component [EC:2.7.1.69]	1
K02823	dihydroorotate dehydrogenase electron transfer subunit	1
K02843	heptosyltransferase II [EC:2.4.-.-]	1
K03091	RNA polymerase sporulation-specific sigma factor	1
K03149	thiamine biosynthesis ThiG	1
K03150	thiamine biosynthesis ThiH	1
K03151	thiamine biosynthesis protein ThiI	1
K03152	4-methyl-5(b-hydroxyethyl)-thiazole monophosphate biosynthesis	1
K03205	type IV secretion system protein VirD4	1
K03216	RNA methyltransferase, TrmH family, group 2 [EC:2.1.1.-]	1
K03271	phosphoheptose isomerase [EC:5.-.-.-]	1
K03272	D-beta-D-heptose 7-phosphate kinase / D-beta-D-heptose 1-phosphate adenosyltransferase [EC:2.7.1.- 2.7.7.-]	1
K03274	ADP-L-glycero-D-manno-heptose 6-epimerase [EC:5.1.3.20]	1
K03286	OmpA-OmpF porin, OOP family	1
K03292	glycoside/pentoside/hexuronide:cation symporter, GPH family	1
K03300	citrate-Mg2+:H+ or citrate-Ca2+:H+ symporter, CitMHS family	1
K03310	alanine or glycine:cation symporter, AGCS family	1
K03311	branched-chain amino acid:cation transporter, LIVCS family	1
K03312	glutamate:Na+ symporter, ESS family	1
K03319	divalent anion:Na+ symporter, DASS family	1
K03325	arsenite transporter, ACR3 family	1
K03386	peroxiredoxin (alkyl hydroperoxide reductase subunit C) [EC:1.11.1.15]	1
K03394	precorrin-2/cobalt-factor-2 C20-methyltransferase [EC:2.1.1.130 2.1.1.151]	1
K03406	methyl-accepting chemotaxis protein	1
K03431	phosphoglucosamine mutase [EC:5.4.2.10]	1
K03438	S-adenosyl-methyltransferase [EC:2.1.1.-]	1
K03471	ribonuclease HIII [EC:3.1.26.4]	1
K03498	trk system potassium uptake protein TrkH	1
K03523	putative biotin biosynthesis protein BioY	1
K03524	BirA family transcriptional regulator, biotin operon repressor / biotin-[acetyl-CoA-carboxylase] ligase [EC:6.3.4.15]	1
K03525	type III pantothenate kinase [EC:2.7.1.33]	1
K03536	ribonuclease P protein component [EC:3.1.26.5]	1
K03543	multidrug resistance protein A	1
K03550	holliday junction DNA helicase RuvA	1
K03564	peroxiredoxin Q/BCP [EC:1.11.1.15]	1
K03565	regulatory protein	1
K03609	septum site-determining protein MinD	1

Continued on next page

Table S2 – Continued from previous page

K03612	electron transport complex protein RnfG	1
K03655	ATP-dependent DNA helicase RecG [EC:3.6.4.12]	1
K03660	N-glycosylase/DNA lyase [EC:3.2.2.- 4.2.99.18]	1
K03665	GTP-binding protein HflX	1
K03686	molecular chaperone DnaJ	1
K03688	ubiquinone biosynthesis protein	1
K03698	CMP-binding protein	1
K03704	cold shock protein (beta-ribbon, CspA family)	1
K03705	heat-inducible transcriptional repressor	1
K03706	transcriptional pleiotropic repressor	1
K03711	Fur family transcriptional regulator, ferric uptake regulator	1
K03734	thiamine biosynthesis lipoprotein	1
K03735	ethanolamine ammonia-lyase large subunit [EC:4.3.1.7]	1
K03741	arsenate reductase [EC:1.20.4.1]	1
K03763	DNA polymerase III subunit alpha, Gram-positive type [EC:2.7.7.7]	1
K03778	D-lactate dehydrogenase [EC:1.1.1.28]	1
K03779	L(+)-tartrate dehydratase alpha subunit [EC:4.2.1.32]	1
K03780	L(+)-tartrate dehydratase beta subunit [EC:4.2.1.32]	1
K03928	carboxylesterase [EC:3.1.1.1]	1
K04041	fructose-1,6-bisphosphatase III [EC:3.1.3.11]	1
K04070	putative pyruvate formate lyase activating enzyme [EC:1.97.1.4]	1
K04485	DNA repair protein RadA/Sms	1
K04486	histidinol-phosphatase (PHP family) [EC:3.1.3.15]	1
K04487	cysteine desulfurase [EC:2.8.1.7]	1
K04720	threonine-phosphate decarboxylase [EC:4.1.1.81]	1
K04767	acetoin utilization protein AcuB	1
K05297	rubredoxin-NAD+ reductase [EC:1.18.1.1]	1
K05311	central glycolytic genes regulator	1
K05685	macrolide transport system ATP-binding/permease protein [EC:3.6.3.-]	1
K05810	conserved hypothetical protein	1
K05832	putative ABC transport system permease protein	1
K05833	putative ABC transport system ATP-binding protein	1
K05837	rod shape determining protein RodA	1
K05896	segregation and condensation protein A	1
K06013	STE24 endopeptidase [EC:3.4.24.84]	1
K06015	N-acyl-D-amino-acid deacylase [EC:3.5.1.81]	1
K06016	N-carbamoyl-L-amino-acid hydrolase [EC:3.5.1.87]	1
K06131	cardiolipin synthase [EC:2.7.8.-]	1
K06147	ATP-binding cassette, subfamily B, bacterial	1
K06148	ATP-binding cassette, subfamily C, bacterial	1
K06200	carbon starvation protein	1
K06201	copper homeostasis protein	1
K06207	GTP-binding protein	1
K06213	magnesium transporter	1
K06223	DNA adenine methylase [EC:2.1.1.72]	1
K06310	spore germination protein	1
K06346	spoIIIJ-associated protein	1
K06390	stage III sporulation protein AA	1

Continued on next page

Table S2 – Continued from previous page

K06399	stage IV sporulation protein B [EC:3.4.21.116]	1
K06405	stage V sporulation protein AC	1
K06409	stage V sporulation protein B	1
K06442	putative hemolysin	1
K06902	MFS transporter, UMF1 family	1
K06958	UPF0042 nucleotide-binding protein	1
K07010	putative glutamine amidotransferase	1
K07023	putative hydrolases of HD superfamily	1
K07160	UPF0271 protein	1
K07166	ACT domain-containing protein	1
K07216	hemerythrin	1
K07240	chromate transporter	1
K07246	tartrate dehydrogenase/decarboxylase / D-malate dehydrogenase [EC:1.1.1.93 4.1.1.73 1.1.1.83]	1
K07258	D-alanyl-D-alanine carboxypeptidase (penicillin-binding protein 5/6) [EC:3.4.16.4]	1
K07279	hypothetical protein	1
K07282	poly-gamma-glutamate synthesis protein (capsule biosynthesis protein)	1
K07301	inner membrane protein	1
K07319	putative adenine-specific DNA-methyltransferase [EC:2.1.1.72]	1
K07396	putative protein-disulfide isomerase	1
K07402	xanthine dehydrogenase accessory factor	1
K07447	putative holliday junction resolvase [EC:3.1.-.-]	1
K07456	DNA mismatch repair protein MutS2	1
K07464	putative RecB family exonuclease	1
K07473	DNA-damage-inducible protein J	1
K07567	TdcF protein	1
K07576	metallo-beta-lactamase family protein	1
K07642	two-component system, OmpR family, sensor histidine kinase BaeS [EC:2.7.13.3]	1
K07657	two-component system, OmpR family, phosphate regulon response regulator PhoB	1
K07665	two-component system, OmpR family, copper resistance phosphate regulon response regulator CusR	1
K07718	two-component system, sensor histidine kinase YesM [EC:2.7.13.3]	1
K07720	two-component system, response regulator YesN	1
K07726	putative transcriptional regulator	1
K07736	CarD family transcriptional regulator	1
K07793	putative tricarboxylic transport membrane protein	1
K07979	GntR family transcriptional regulator	1
K08384	stage V sporulation protein D (sporulation-specific penicillin-binding protein)	1
K08884	serine/threonine protein kinase, bacterial [EC:2.7.11.1]	1
K08974	putative membrane protein	1
K09137	hypothetical protein	1
K09685	purine operon repressor	1
K09687	antibiotic transport system ATP-binding protein	1
K09698	nondiscriminating glutamyl-tRNA synthetase [EC:6.1.1.24]	1

Continued on next page

Table S2 – Continued from previous page

K09729	hypothetical protein	1
K09759	nondiscriminating aspartyl-tRNA synthetase [EC:6.1.1.23]	1
K09762	hypothetical protein	1
K09767	hypothetical protein	1
K09773	hypothetical protein	1
K09788	hypothetical protein	1
K09800	hypothetical protein	1
K09815	zinc transport system substrate-binding protein	1
K09824	hypothetical protein	1
K09825	Fur family transcriptional regulator, peroxide stress response regulator	1
K09861	hypothetical protein	1
K09949	hypothetical protein	1
K10036	glutamine transport system substrate-binding protein	1
K10040	putative glutamine transport system permease protein	1
K10108	maltose/maltodextrin transport system substrate-binding protein	1
K10109	maltose/maltodextrin transport system permease protein	1
K10110	maltose/maltodextrin transport system permease protein	1
K10117	multiple sugar transport system substrate-binding protein	1
K10118	multiple sugar transport system permease protein	1
K10119	multiple sugar transport system permease protein	1
K10188	lactose/L-arabinose transport system substrate-binding protein	1
K10541	methyl-galactoside transport system permease protein	1
K10806	acyl-CoA thioesterase YciA [EC:3.1.2.-]	1
K11065	thiol peroxidase, atypical 2-Cys peroxiredoxin [EC:1.11.1.15]	1
K11069	spermidine/putrescine transport system substrate-binding protein	1
K11070	spermidine/putrescine transport system permease protein	1
K11071	spermidine/putrescine transport system permease protein	1
K11072	spermidine/putrescine transport system ATP-binding protein [EC:3.6.3.31]	1
K11358	aspartate aminotransferase [EC:2.6.1.1]	1
K11755	phosphoribosyl-ATP pyrophosphohydrolase / phosphoribosyl-AMP cyclohydrolase [EC:3.6.1.31 3.5.4.19]	1
K11900	type VI secretion system protein ImpC	1
K11907	type VI secretion system protein VasG	1
K12308	beta-galactosidase [EC:3.2.1.23]	1
K12506	2-C-methyl-D-erythritol 4-phosphate cytidylyltransferase / 2-C-methyl-D-erythritol 2,4-cyclodiphosphate synthase [EC:2.7.7.60 4.6.1.12]	1
K12574	ribonuclease J [EC:3.1.-.-]	1
K12940	aminobenzoyl-glutamate utilization protein A	1
K12941	aminobenzoyl-glutamate utilization protein B	1
K12942	aminobenzoyl-glutamate transport protein	1
K13049	carboxypeptidase PM20D1 [EC:3.4.17.-]	1
K13685	UDP-N-acetylglucosamine:undecaprenyl-P N-acetylglucosaminyl 1-P transferase [EC:2.7.8.-]	1

Continued on next page

K13927	holo-ACP synthase / triphosphoribosyl-dephospho-CoA synthase [EC:2.7.7.61 2.7.8.25]	1
K13929	malonate decarboxylase alpha subunit [EC:2.3.1.187]	1
K14170	chorismate mutase / prephenate dehydratase [EC:5.4.99.5 4.2.1.51]	1
K14260	alanine-synthesizing transaminase [EC:2.6.1.66 2.6.1.2]	1
K14540	ribosome biogenesis GTPase A	1
K15035	transfer-messenger RNA	1
K15051	DNA-entry nuclease	1
K00014	shikimate dehydrogenase [EC:1.1.1.25]	2
K00075	UDP-N-acetylmuramate dehydrogenase [EC:1.1.1.158]	2
K00147	glutamate-5-semialdehyde dehydrogenase [EC:1.2.1.41]	2
K00243	hypothetical protein	2
K00286	pyrroline-5-carboxylate reductase [EC:1.5.1.2]	2
K00297	methylenetetrahydrofolate reductase (NADPH) [EC:1.5.1.20]	2
K00375	GntR family transcriptional regulator / MocR family aminotransferase	2
K00394	adenylylsulfate reductase, subunit A [EC:1.8.99.2]	2
K00548	5-methyltetrahydrofolate--homocysteine methyltransferase [EC:2.1.1.13]	2
K00566	tRNA-specific 2-thiouridylase [EC:2.8.1.-]	2
K00620	glutamate N-acetyltransferase / amino-acid N-acetyltransferase [EC:2.3.1.35 2.3.1.1]	2
K00655	1-acyl-sn-glycerol-3-phosphate acyltransferase [EC:2.3.1.51]	2
K00703	starch synthase [EC:2.4.1.21]	2
K00783	hypothetical protein	2
K00790	UDP-N-acetylglucosamine 1-carboxyvinyltransferase [EC:2.5.1.7]	2
K00793	riboflavin synthase alpha chain [EC:2.5.1.9]	2
K00859	dephospho-CoA kinase [EC:2.7.1.24]	2
K00868	pyridoxine kinase [EC:2.7.1.35]	2
K00930	acetylglutamate/acetylaminoadipate kinase [EC:2.7.2.8 2.7.2.-]	2
K00941	hydroxymethylpyrimidine/phosphomethylpyrimidine kinase [EC:2.7.1.49 2.7.4.7]	2
K00956	sulfate adenylyltransferase subunit 1 [EC:2.7.7.4]	2
K00957	sulfate adenylyltransferase subunit 2 [EC:2.7.7.4]	2
K00969	nicotinate-nucleotide adenylyltransferase [EC:2.7.7.18]	2
K00972	UDP-N-acetylglucosamine pyrophosphorylase [EC:2.7.7.23]	2
K00974	tRNA nucleotidyltransferase (CCA-adding enzyme) [EC:2.7.7.72 3.1.3.- 3.1.4.-]	2
K00981	phosphatidate cytidylyltransferase [EC:2.7.7.41]	2
K00998	phosphatidylserine synthase [EC:2.7.8.8]	2
K01000	phospho-N-acetylmuramoyl-pentapeptide-transferase [EC:2.7.8.13]	2
K01012	biotin synthetase [EC:2.8.1.6]	2
K01056	peptidyl-tRNA hydrolase, PTH1 family [EC:3.1.1.29]	2
K01142	exodeoxyribonuclease III [EC:3.1.11.2]	2
K01269	aminopeptidase [EC:3.4.11.-]	2

Continued on next page

Table S2 – Continued from previous page

K01356	repressor LexA [EC:3.4.21.88]	2
K01439	succinyl-diaminopimelate desuccinylase [EC:3.5.1.18]	2
K01462	peptide deformylase [EC:3.5.1.88]	2
K01465	dihydroorotase [EC:3.5.2.3]	2
K01493	dCMP deaminase [EC:3.5.4.12]	2
K01647	citrate synthase [EC:2.3.3.1]	2
K01652	acetolactate synthase I/II/III large subunit [EC:2.2.1.6]	2
K01657	anthranilate synthase component I [EC:4.1.3.27]	2
K01662	1-deoxy-D-xylulose-5-phosphate synthase [EC:2.2.1.7]	2
K01666	4-hydroxy 2-oxovalerate aldolase [EC:4.1.3.39]	2
K01681	aconitate hydratase 1 [EC:4.2.1.3]	2
K01687	dihydroxy-acid dehydratase [EC:4.2.1.9]	2
K01736	chorismate synthase [EC:4.2.3.5]	2
K01814	phosphoribosylformimino-5-aminoimidazole carboxamide ribotide isomerase [EC:5.3.1.16]	2
K01924	UDP-N-acetylmuramate--alanine ligase [EC:6.3.2.8]	2
K01928	UDP-N-acetylmuramoylalanyl-D-glutamate--2,6-diaminopimelate ligase [EC:6.3.2.13]	2
K01934	5-formyltetrahydrofolate cyclo-ligase [EC:6.3.3.2]	2
K01956	carbamoyl-phosphate synthase small subunit [EC:6.3.5.5]	2
K01966	propionyl-CoA carboxylase beta chain [EC:6.4.1.3]	2
K02006	cobalt/nickel transport system ATP-binding protein	2
K02016	iron complex transport system substrate-binding protein	2
K02045	sulfate transport system ATP-binding protein [EC:3.6.3.25]	2
K02047	sulfate transport system permease protein	2
K02048	sulfate transport system substrate-binding protein	2
K02071	D-methionine transport system ATP-binding protein	2
K02072	D-methionine transport system permease protein	2
K02073	D-methionine transport system substrate-binding protein	2
K02203	phosphoserine / homoserine phosphotransferase [EC:3.1.3.3 2.7.1.39]	2
K02313	chromosomal replication initiator protein	2
K02338	DNA polymerase III subunit beta [EC:2.7.7.7]	2
K02341	DNA polymerase III subunit delta [EC:2.7.7.7]	2
K02372	3R-hydroxymyristoyl ACP dehydrase [EC:4.2.1.-]	2
K02517	lipid A biosynthesis lauroyl acyltransferase [EC:2.3.1.-]	2
K02654	leader peptidase (prepilin peptidase) / N-methyltransferase [EC:3.4.23.43 2.1.1.-]	2
K02669	twitching motility protein PilT	2
K02687	ribosomal protein L11 methyltransferase [EC:2.1.1.-]	2
K03073	preprotein translocase subunit SecE	2
K03154	sulfur carrier protein	2
K03218	RNA methyltransferase, TrmH family [EC:2.1.1.-]	2
K03426	NAD+ diphosphatase [EC:3.6.1.22]	2
K03437	RNA methyltransferase, TrmH family	2
K03442	small conductance mechanosensitive channel	2
K03453	bile acid:Na+ symporter, BASS family	2
K03458	nucleobase:cation symporter-2, NCS2 family	2
K03466	DNA segregation ATPase FtsK/SpoIIIE, S-DNA-T family	2
K03469	ribonuclease HI [EC:3.1.26.4]	2
K03470	ribonuclease HII [EC:3.1.26.4]	2

Continued on next page

K03495	glucose inhibited division protein A	2
K03500	ribosomal RNA small subunit methyltransferase B [EC:2.1.1.-]	2
K03529	chromosome segregation protein	2
K03546	exonuclease SbcC	2
K03581	exodeoxyribonuclease V alpha subunit [EC:3.1.11.5]	2
K03601	exodeoxyribonuclease VII large subunit [EC:3.1.11.6]	2
K03621	glycerol-3-phosphate acyltransferase PlsX [EC:2.3.1.15]	2
K03629	DNA replication and repair protein RecF	2
K03631	DNA repair protein RecN (Recombination protein N)	2
K03650	tRNA modification GTPase	2
K03657	DNA helicase II / ATP-dependent DNA helicase PcrA [EC:3.6.4.12]	2
K03664	SsrA-binding protein	2
K03671	thioredoxin 1	2
K03703	excinuclease ABC subunit C	2
K03723	transcription-repair coupling factor (superfamily II helicase) [EC:3.6.4.-]	2
K03785	3-dehydroquinate dehydratase I [EC:4.2.1.10]	2
K03816	xanthine phosphoribosyltransferase [EC:2.4.2.22]	2
K04066	primosomal protein N (replication factor Y) (superfamily II helicase) [EC:3.6.4.-]	2
K04075	tRNA(Ile)-lysidine synthase [EC:6.3.4.-]	2
K04083	molecular chaperone Hsp33	2
K04758	ferrous iron transport protein A	2
K04763	integrase/recombinase XerD	2
K06023	HPr kinase/phosphorylase [EC:2.7.11.- 2.7.4.-]	2
K06133	4 -phosphopantetheinyl transferase [EC:2.7.8.-]	2
K06158	ATP-binding cassette, sub-family F, member 3	2
K06168	bifunctional enzyme involved in thiolation and methylation of tRNA	2
K06173	tRNA pseudouridine synthase A [EC:5.4.99.12]	2
K06179	ribosomal large subunit pseudouridine synthase C [EC:5.4.99.12]	2
K06183	ribosomal small subunit pseudouridine synthase A [EC:5.4.99.12]	2
K06187	recombination protein RecR	2
K06287	septum formation protein	2
K06381	stage II sporulation protein D	2
K06518	holin-like protein	2
K06925	UPF0079 ATP-binding protein	2
K06969	ribosomal RNA large subunit methyltransferase I [EC:2.1.1.-]	2
K07040	uncharacterized protein	2
K07082	UPF0755 protein	2
K07238	zinc transporter, ZIP family	2
K07460	putative endonuclease	2
K07461	putative endonuclease	2
K07738	transcriptional repressor NrdR	2
K08303	putative protease [EC:3.4.-.-]	2
K08483	phosphotransferase system, enzyme I, PtsI [EC:2.7.3.9]	2

Continued on next page

Table S2 – Continued from previous page

K08591	glycerol-3-phosphate acyltransferase PlsY [EC:2.3.1.15]	2
K08602	oligoendopeptidase F [EC:3.4.24.-]	2
K08681	glutamine amidotransferase [EC:2.6.-.-]	2
K09117	hypothetical protein	2
K09686	antibiotic transport system permease protein	2
K09710	hypothetical protein	2
K09761	ribosomal RNA small subunit methyltransferase E [EC:2.1.1.-]	2
K09765	hypothetical protein	2
K09811	cell division transport system permease protein	2
K09816	zinc transport system permease protein	2
K09817	zinc transport system ATP-binding protein [EC:3.6.3.-]	2
K09951	CRISPR-associated protein Cas2	2
K10536	agmatine deiminase [EC:3.5.3.12]	2
K10907	aminotransferase [EC:2.6.1.-]	2
K10914	CRP/FNR family transcriptional regulator, cyclic AMP receptor protein	2
K11189	phosphocarrier protein	2
K11752	diaminohydroxyphosphoribosylaminopyrimidine deaminase / 5-amino-6-(5-phosphoribosylamino)uracil reductase [EC:3.5.4.26 1.1.1.193]	2
K11753	riboflavin kinase / FMN adenylyltransferase [EC:2.7.1.26 2.7.7.2]	2
K11754	dihydrofolate synthase / folylpolyglutamate synthase [EC:6.3.2.12 6.3.2.17]	2
K11928	sodium/proline symporter	2
K12410	NAD-dependent deacetylase [EC:3.5.1.-]	2
K13292	phosphatidylglycerol:prolipoprotein diacylglycerol transferase [EC:2.-.-.-]	2
K13542	uroporphyrinogen III methyltransferase / synthase [EC:2.1.1.107 4.2.1.75]	2
K14155	cystathione beta-lyase [EC:4.4.1.8]	2
K14441	ribosomal protein S12 methylthiotransferase [EC:2.-.-.-]	2
K00012	UDPglucose 6-dehydrogenase [EC:1.1.1.22]	3
K00018	glycerate dehydrogenase [EC:1.1.1.29]	3
K00259	alanine dehydrogenase [EC:1.4.1.1]	3
K00282	glycine dehydrogenase subunit 1 [EC:1.4.4.2]	3
K00426	cytochrome bd-I oxidase subunit II [EC:1.10.3.-]	3
K00602	phosphoribosylaminoimidazolecarboxamide formyltransferase / IMP cyclohydrolase [EC:2.1.2.3 3.5.4.10]	3
K00606	3-methyl-2-oxobutanoate hydroxymethyltransferase [EC:2.1.2.11]	3
K00610	aspartate carbamoyltransferase regulatory subunit	3
K00648	3-oxoacyl-[acyl-carrier-protein] synthase III [EC:2.3.1.180]	3
K00684	leucyl/phenylalanyl-tRNA--protein transferase [EC:2.3.2.6]	3
K00757	uridine phosphorylase [EC:2.4.2.3]	3
K00782	hypothetical protein	3
K00806	undecaprenyl diphosphate synthase [EC:2.5.1.31]	3
K00811	aspartate aminotransferase [EC:2.6.1.1]	3
K00818	acetylornithine aminotransferase [EC:2.6.1.11]	3
K00848	rhamnulokinase [EC:2.7.1.5]	3

Continued on next page

Table S2 – Continued from previous page

K00857	thymidine kinase [EC:2.7.1.21]	3
K00876	uridine kinase [EC:2.7.1.48]	3
K00897	aminoglycoside 3 -phosphotransferase [EC:2.7.1.95]	3
K00950	2-amino-4-hydroxy-6-hydroxymethyldihydropteridine diphosphokinase [EC:2.7.6.3]	3
K00971	mannose-1-phosphate guanylyltransferase [EC:2.7.7.22]	3
K00979	3-deoxy-manno-octulosonate cytidylyltransferase (CMP-KDO synthetase) [EC:2.7.7.38]	3
K01051	pectinesterase [EC:3.1.1.11]	3
K01085	glucose-1-phosphatase [EC:3.1.3.10]	3
K01119	2 ,3 -cyclic-nucleotide 2 -phosphodiesterase [EC:3.1.4.16]	3
K01154	type I restriction enzyme, S subunit [EC:3.1.21.3]	3
K01185	lysozyme [EC:3.2.1.17]	3
K01191	alpha-mannosidase [EC:3.2.1.24]	3
K01206	alpha-L-fucosidase [EC:3.2.1.51]	3
K01243	S-adenosylhomocysteine/5 -methylthioadenosine nucleosidase [EC:3.2.2.9]	3
K01256	aminopeptidase N [EC:3.4.11.2]	3
K01277	dipeptidyl-peptidase III [EC:3.4.14.4]	3
K01278	dipeptidyl-peptidase 4 [EC:3.4.14.5]	3
K01297	muramoyltetrapeptide carboxypeptidase [EC:3.4.17.13]	3
K01372	bleomycin hydrolase [EC:3.4.22.40]	3
K01438	acetylornithine deacetylase [EC:3.5.1.16]	3
K01489	cytidine deaminase [EC:3.5.4.5]	3
K01501	nitrilase [EC:3.5.5.1]	3
K01520	dUTP pyrophosphatase [EC:3.6.1.23]	3
K01599	uroporphyrinogen decarboxylase [EC:4.1.1.37]	3
K01619	deoxyribose-phosphate aldolase [EC:4.1.2.4]	3
K01711	GDPmannose 4,6-dehydratase [EC:4.2.1.47]	3
K01713	prephenate dehydratase [EC:4.2.1.51]	3
K01719	uroporphyrinogen-III synthase [EC:4.2.1.75]	3
K01776	glutamate racemase [EC:5.1.1.3]	3
K01785	aldose 1-epimerase [EC:5.1.3.3]	3
K01790	dTDP-4-dehydrorhamnose 3,5-epimerase [EC:5.1.3.13]	3
K01809	mannose-6-phosphate isomerase [EC:5.3.1.8]	3
K01897	long-chain acyl-CoA synthetase [EC:6.2.1.3]	3
K01911	O-succinylbenzoic acid--CoA ligase [EC:6.2.1.26]	3
K01914	aspartate--ammonia ligase [EC:6.3.1.1]	3
K01990	ABC-2 type transport system ATP-binding protein	3
K02005	HlyD family secretion protein	3
K02014	iron complex outermembrane recepter protein	3
K02069	putative ABC transport system permease protein	3
K02335	DNA polymerase I [EC:2.7.7.7]	3
K02340	DNA polymerase III subunit delta [EC:2.7.7.7]	3
K02346	DNA polymerase IV [EC:2.7.7.7]	3
K02361	isochorismate synthase [EC:5.4.4.2]	3
K02429	MFS transporter, FHS family, L-fucose permease	3
K02437	glycine cleavage system H protein	3
K02549	O-succinylbenzoate synthase [EC:4.2.1.113]	3
K02551	2-succinyl-5-enolpyruvyl-6-hydroxy-3-cyclohexene-1-carboxylate synthase [EC:2.2.1.9]	3

Continued on next page

K02836	peptide chain release factor RF-2	3
K02860	16S rRNA processing protein RimM	3
K02897	large subunit ribosomal protein L25	3
K02916	large subunit ribosomal protein L35	3
K03075	preprotein translocase subunit SecG	3
K03116	sec-independent protein translocase protein TatA	3
K03118	sec-independent protein translocase protein TatC	3
K03210	preprotein translocase subunit YajC	3
K03269	UDP-2,3-diacylglucosamine hydrolase [EC:3.6.1.-]	3
K03270	3-deoxy-D-manno-octulosonate 8-phosphate phosphatase (KDO 8-P phosphatase) [EC:3.1.3.45]	3
K03308	neurotransmitter:Na+ symporter, NSS family	3
K03315	Na+:H+ antiporter, NhaC family	3
K03402	transcriptional regulator of arginine metabolism	3
K03455	monovalent cation:H+ antiporter-2, CPA2 family	3
K03534	L-rhamnose mutarotase [EC:5.1.3.-]	3
K03602	exodeoxyribonuclease VII small subunit [EC:3.1.11.6]	3
K03606	putative colanic acid biosysnthesis UDP-glucose lipid carrier transferase	3
K03625	N utilization substance protein B	3
K03630	DNA repair protein RadC	3
K03685	ribonuclease III [EC:3.1.26.3]	3
K03718	Lrp/AsnC family transcriptional regulator, regulator for asnA, asnC and gidA	3
K03733	integrase/recombinase XerC	3
K03742	competence/damage-inducible protein CinA	3
K03773	FKBP-type peptidyl-prolyl cis-trans isomerase FklB [EC:5.2.1.8]	3
K03786	3-dehydroquinate dehydratase II [EC:4.2.1.10]	3
K03811	nicotinamide mononucleotide transporter	3
K03979	GTP-binding protein	3
K04069	pyruvate formate lyase activating enzyme [EC:1.97.1.4]	3
K04516	chorismate mutase [EC:5.4.99.5]	3
K04751	nitrogen regulatory protein P-II 1	3
K04764	integration host factor subunit alpha	3
K05499	LacI family transcriptional regulator, repressor for deo operon, udp, cdd, tsx, nupC, and nupG	3
K05593	aminoglycoside 6-adenylyltransferase [EC:2.7.7.-]	3
K05794	tellurite resistance protein TerC	3
K05970	sialate O-acetylesterase [EC:3.1.1.53]	3
K06041	arabinose-5-phosphate isomerase [EC:5.3.1.13]	3
K06076	long-chain fatty acid transport protein	3
K06113	arabinan endo-1,5-alpha-L-arabinosidase [EC:3.2.1.99]	3
K06167	PhnP protein	3
K06177	ribosomal large subunit pseudouridine synthase A [EC:5.4.99.12]	3
K06180	ribosomal large subunit pseudouridine synthase D [EC:5.4.99.12]	3
K06188	aquaporin Z	3
K07085	putative transport protein	3
K07106	N-acetylmuramic acid 6-phosphate etherase [EC:4.2.-.-]	3

Continued on next page

Table S2 – Continued from previous page

K07114	uncharacterized protein	3
K07263	zinc protease [EC:3.4.99.-]	3
K07271	lipopolysaccharide cholinephosphotransferase [EC:2.7.8.-]	3
K07273	lysozyme	3
K07334	proteic killer suppression protein	3
K07444	putative N6-adenine-specific DNA methylase [EC:2.1.1.-]	3
K07462	single-stranded-DNA-specific exonuclease [EC:3.1.-.-]	3
K07636	two-component system, OmpR family, phosphate regulon sensor histidine kinase PhoR [EC:2.7.13.3]	3
K07737	putative transcriptional regulator	3
K07791	anaerobic C4-dicarboxylate transporter DcuA	3
K08218	MFS transporter, PAT family, beta-lactamase induction signal transducer AmpG	3
K09125	hypothetical protein	3
K09680	type II pantothenate kinase [EC:2.7.1.33]	3
K09704	hypothetical protein	3
K09973	hypothetical protein	3
K10947	PadR family transcriptional regulator, regulatory protein PadR	3
K11068	hemolysin III	3
K11921	LysR family transcriptional regulator	3
K12373	hexosaminidase [EC:3.2.1.52]	3
K00046	gluconate 5-dehydrogenase [EC:1.1.1.69]	4
K00123	formate dehydrogenase, alpha subunit [EC:1.2.1.2]	4
K00125	formate dehydrogenase, beta subunit [EC:1.2.1.2]	4
K00150	glyceraldehyde-3-phosphate dehydrogenase (NAD(P)) [EC:1.2.1.59]	4
K00169	pyruvate ferredoxin oxidoreductase, alpha subunit [EC:1.2.7.1]	4
K00170	pyruvate ferredoxin oxidoreductase, beta subunit [EC:1.2.7.1]	4
K00187	2-oxoisovalerate ferredoxin oxidoreductase, beta subunit [EC:1.2.7.7]	4
K00200	formylmethanofuran dehydrogenase subunit A [EC:1.2.99.5]	4
K00201	formylmethanofuran dehydrogenase subunit B [EC:1.2.99.5]	4
K00202	formylmethanofuran dehydrogenase subunit C [EC:1.2.99.5]	4
K00203	formylmethanofuran dehydrogenase subunit D [EC:1.2.99.5]	4
K00205	formylmethanofuran dehydrogenase subunit F [EC:1.2.99.5]	4
K00399	methyl-coenzyme M reductase alpha subunit [EC:2.8.4.1]	4
K00400	methyl coenzyme M reductase system, component A2	4
K00401	methyl-coenzyme M reductase beta subunit [EC:2.8.4.1]	4
K00402	methyl-coenzyme M reductase gamma subunit [EC:2.8.4.1]	4
K00440	coenzyme F420 hydrogenase alpha subunit [EC:1.12.98.1]	4
K00441	coenzyme F420 hydrogenase beta subunit [EC:1.12.98.1]	4
K00443	coenzyme F420 hydrogenase gamma subunit [EC:1.12.98.1]	4

Continued on next page

Table S2 – Continued from previous page

K00577	tetrahydromethanopterin S-methyltransferase subunit A [EC:2.1.1.86]	4
K00580	tetrahydromethanopterin S-methyltransferase subunit D [EC:2.1.1.86]	4
K00581	tetrahydromethanopterin S-methyltransferase subunit E [EC:2.1.1.86]	4
K00584	tetrahydromethanopterin S-methyltransferase subunit H [EC:2.1.1.86]	4
K00638	chloramphenicol O-acetyltransferase [EC:2.3.1.28]	4
K00672	formylmethanofuran--tetrahydromethanopterin N-formyltransferase [EC:2.3.1.101]	4
K00940	nucleoside-diphosphate kinase [EC:2.7.4.6]	4
K01007	pyruvate, water dikinase [EC:2.7.9.2]	4
K01079	phosphoserine phosphatase [EC:3.1.3.3]	4
K01499	methenyltetrahydromethanopterin cyclohydrolase [EC:3.5.4.27]	4
K01622	fructose 1,6-bisphosphate aldolase/phosphatase [EC:4.1.2.13 3.1.3.11]	4
K01623	fructose-bisphosphate aldolase, class I [EC:4.1.2.13]	4
K01823	isopentenyl-diphosphate delta-isomerase [EC:5.3.3.2]	4
K01959	pyruvate carboxylase subunit A [EC:6.4.1.1]	4
K02007	cobalt/nickel transport system permease protein	4
K02009	cobalt transport protein	4
K02030	polar amino acid transport system substrate-binding protein	4
K02118	V-type H+-transporting ATPase subunit B [EC:3.6.3.14]	4
K02119	V-type H+-transporting ATPase subunit C [EC:3.6.3.14]	4
K02121	V-type H+-transporting ATPase subunit E [EC:3.6.3.14]	4
K02122	V-type H+-transporting ATPase subunit F [EC:3.6.3.14]	4
K02123	V-type H+-transporting ATPase subunit I [EC:3.6.3.14]	4
K02124	V-type H+-transporting ATPase subunit K [EC:3.6.3.14]	4
K02217	ferritin [EC:1.16.3.1]	4
K02319	DNA polymerase I [EC:2.7.7.7]	4
K02322	DNA polymerase II large subunit [EC:2.7.7.7]	4
K02323	DNA polymerase II small subunit [EC:2.7.7.7]	4
K02585	nitrogen fixation protein NifB	4
K02877	large subunit ribosomal protein L15e	4
K02885	large subunit ribosomal protein L19e	4
K02908	large subunit ribosomal protein L30e	4
K02912	large subunit ribosomal protein L32e	4
K02915	large subunit ribosomal protein L34e	4
K02930	large subunit ribosomal protein L4e	4
K02979	small subunit ribosomal protein S28e	4
K02984	small subunit ribosomal protein S3Ae	4
K02987	small subunit ribosomal protein S4e	4
K03041	DNA-directed RNA polymerase subunit A [EC:2.7.7.6]	4
K03042	DNA-directed RNA polymerase subunit A [EC:2.7.7.6]	4
K03044	DNA-directed RNA polymerase subunit B [EC:2.7.7.6]	4
K03045	DNA-directed RNA polymerase subunit B [EC:2.7.7.6]	4
K03047	DNA-directed RNA polymerase subunit D [EC:2.7.7.6]	4
K03049	DNA-directed RNA polymerase subunit E [EC:2.7.7.6]	4
K03113	translation initiation factor eIF-1	4

Continued on next page

K03120	transcription initiation factor TFIID TATA-box-binding protein	4
K03124	transcription initiation factor TFIIB	4
K03166	DNA topoisomerase VI subunit A [EC:5.99.1.3]	4
K03167	DNA topoisomerase VI subunit B [EC:5.99.1.3]	4
K03234	elongation factor EF-2 [EC:3.6.5.3]	4
K03236	translation initiation factor eIF-1A	4
K03237	translation initiation factor eIF-2 alpha subunit	4
K03242	translation initiation factor eIF-2 gamma subunit	4
K03265	peptide chain release factor eRF subunit 1	4
K03388	heterodisulfide reductase subunit A [EC:1.8.98.1]	4
K03390	heterodisulfide reductase subunit C [EC:1.8.98.1]	4
K03420	proteasome regulatory subunit	4
K03421	methyl-coenzyme M reductase subunit C	4
K03432	proteasome alpha subunit [EC:3.4.25.1]	4
K03433	proteasome beta subunit [EC:3.4.25.1]	4
K03531	cell division protein FtsZ	4
K03679	exosome complex component RRP4	4
K03750	molybdopterin biosynthesis protein MoeA	4
K04076	Lon-like ATP-dependent protease [EC:3.4.21.-]	4
K04483	DNA repair protein RadA	4
K04566	lysyl-tRNA synthetase, class I [EC:6.1.1.6]	4
K04654	hydrogenase expression/formation protein HypD	4
K04655	hydrogenase expression/formation protein HypE	4
K04656	hydrogenase maturation protein HypF	4
K05884	(R)-2-hydroxyacid dehydrogenase [EC:1.1.1.272]	4
K06042	precorrin-8X methylmutase [EC:5.4.1.2]	4
K06174	ATP-binding cassette, sub-family E, member 1	4
K06863	5-formaminoimidazole-4-carboxamide-1-(beta)-D-ribofuranosyl 5 -monophosphate synthetase [EC:6.3.4.-]	4
K07388	hydrogenase expression/formation protein	4
K07463	archaea-specific RecJ-like exonuclease	4
K07574	putative RNA-binding protein containing KH domain	4
K08096	GTP cyclohydrolase IIa [EC:3.5.4.29]	4
K08963	methylthioribose-1-phosphate isomerase [EC:5.3.1.23]	4
K09123	hypothetical protein	4
K09142	hypothetical protein	4
K09154	hypothetical protein	4
K09181	hypothetical protein	4
K09482	glutamyl-tRNA(Gln) amidotransferase subunit D [EC:6.3.5.7]	4
K09726	hypothetical protein	4
K10725	archaeal cell division control protein 6	4
K10726	replicative DNA helicase Mcm [EC:3.6.4.-]	4
K10747	DNA ligase 1 [EC:6.5.1.1]	4
K11600	exosome complex component RRP41	4
K11780	FO synthase subunit 1 [EC:2.5.1.-]	4
K13525	transitional endoplasmic reticulum ATPase	4
K13812	bifunctional enzyme Fae/Hps [EC:4.3.-.- 4.1.2.43]	4
K13942	5,10-methenyltetrahydromethanopterin hydrogenase [EC:1.12.98.2]	4

Continued on next page

Table S2 – Continued from previous page

K14101	energy-converting hydrogenase A subunit J	4
K14105	energy-converting hydrogenase A subunit N	4
K14106	energy-converting hydrogenase A subunit O	4
K14123	energy-converting hydrogenase B subunit N	4
K14126	F420-non-reducing hydrogenase subunit A [EC:1.12.99.-]	4
K14128	F420-non-reducing hydrogenase subunit G [EC:1.12.99.-]	4
K00001	alcohol dehydrogenase [EC:1.1.1.1]	5
K00003	homoserine dehydrogenase [EC:1.1.1.3]	5
K00029	malate dehydrogenase (oxaloacetate-decarboxylating)(NADP+) [EC:1.1.1.40]	5
K00067	dTDP-4-dehydrorhamnose reductase [EC:1.1.1.133]	5
K00077	2-dehydropantoate 2-reductase [EC:1.1.1.169]	5
K00128	aldehyde dehydrogenase (NAD+) [EC:1.2.1.3]	5
K00336	NADH dehydrogenase I subunit G [EC:1.6.5.3]	5
K00337	NADH dehydrogenase I subunit H [EC:1.6.5.3]	5
K00343	NADH dehydrogenase I subunit N [EC:1.6.5.3]	5
K00549	5-methyltetrahydropteroyltriglutamate--homocysteine methyltransferase [EC:2.1.1.14]	5
K00554	tRNA (guanine-N1-)-methyltransferase [EC:2.1.1.31]	5
K00567	methylated-DNA-[protein]-cysteine S-methyltransferase [EC:2.1.1.63]	5
K00767	nicotinate-nucleotide pyrophosphorylase (carboxylating) [EC:2.4.2.19]	5
K00773	queuine tRNA-ribosyltransferase [EC:2.4.2.29]	5
K00796	dihydropteroate synthase [EC:2.5.1.15]	5
K00817	histidinol-phosphate aminotransferase [EC:2.6.1.9]	5
K00833	adenosylmethionine-8-amino-7-oxononanoate aminotransferase [EC:2.6.1.62]	5
K00989	ribonuclease PH [EC:2.7.7.56]	5
K00995	CDP-diacylglycerol--glycerol-3-phosphate 3-phosphatidyltransferase [EC:2.7.8.5]	5
K01092	myo-inositol-1(or 4)-monophosphatase [EC:3.1.3.25]	5
K01104	protein-tyrosine phosphatase [EC:3.1.3.48]	5
K01129	dGTPase [EC:3.1.5.1]	5
K01159	crossover junction endodeoxyribonuclease RuvC [EC:3.1.22.4]	5
K01179	endoglucanase [EC:3.2.1.4]	5
K01207	beta-N-acetylhexosaminidase [EC:3.2.1.52]	5
K01224	arabinogalactan endo-1,4-beta-galactosidase [EC:3.2.1.89]	5
K01424	L-asparaginase [EC:3.5.1.1]	5
K01572	oxaloacetate decarboxylase, beta subunit [EC:4.1.1.3]	5
K01579	aspartate 1-decarboxylase [EC:4.1.1.11]	5
K01613	phosphatidylserine decarboxylase [EC:4.1.1.65]	5
K01689	enolase [EC:4.2.1.11]	5
K01693	imidazoleglycerol-phosphate dehydratase [EC:4.2.1.19]	5
K01698	porphobilinogen synthase [EC:4.2.1.24]	5
K01745	histidine ammonia-lyase [EC:4.3.1.3]	5
K01760	cystathionine beta-lyase [EC:4.4.1.8]	5
K01807	ribose 5-phosphate isomerase A [EC:5.3.1.6]	5
K01845	glutamate-1-semialdehyde 2,1-aminomutase [EC:5.4.3.8]	5
K01915	glutamine synthetase [EC:6.3.1.2]	5

Continued on next page

Table S2 – Continued from previous page

K01993	HlyD family secretion protein	5
K02050	sulfonate/nitrate/taurine transport system permease protein	5
K02065	putative ABC transport system ATP-binding protein	5
K02066	putative ABC transport system permease protein	5
K02067	putative ABC transport system substrate-binding protein	5
K02237	competence protein ComEA	5
K02238	competence protein ComEC	5
K02343	DNA polymerase III subunit gamma/tau [EC:2.7.7.7]	5
K02454	general secretion pathway protein E	5
K02492	glutamyl-tRNA reductase [EC:1.2.1.70]	5
K02586	nitrogenase molybdenum-iron protein alpha chain [EC:1.18.6.1]	5
K02588	nitrogenase iron protein NifH [EC:1.18.6.1]	5
K02591	nitrogenase molybdenum-iron protein beta chain [EC:1.18.6.1]	5
K02652	type IV pilus assembly protein PilB	5
K03092	RNA polymerase sigma-54 factor	5
K03101	signal peptidase II [EC:3.4.23.36]	5
K03111	single-strand DNA-binding protein	5
K03169	DNA topoisomerase III [EC:5.99.1.2]	5
K03183	ubiquinone/menaquinone biosynthesis methyltransferase [EC:2.1.1.-]	5
K03296	hydrophobic/amphiphilic exporter-1 (mainly G- bacteria), HAE1 family	5
K03321	sulfate permease, SulP family	5
K03327	multidrug resistance protein, MATE family	5
K03424	TatD DNase family protein [EC:3.1.21.-]	5
K03439	tRNA (guanine-N7-)-methyltransferase [EC:2.1.1.33]	5
K03501	glucose inhibited division protein B [EC:2.1.-.-]	5
K03559	biopolymer transport protein ExbD	5
K03570	rod shape-determining protein MreC	5
K03574	7,8-dihydro-8-oxoguanine triphosphatase [EC:3.6.1.-]	5
K03589	cell division protein FtsQ	5
K03593	ATP-binding protein involved in chromosome partitioning	5
K03687	molecular chaperone GrpE	5
K03744	LemA protein	5
K03770	peptidyl-prolyl cis-trans isomerase D [EC:5.2.1.8]	5
K03771	peptidyl-prolyl cis-trans isomerase SurA [EC:5.2.1.8]	5
K03787	5 -nucleotidase [EC:3.1.3.5]	5
K03799	heat shock protein HtpX [EC:3.4.24.-]	5
K03924	MoxR-like ATPase [EC:3.6.3.-]	5
K04094	glucose inhibited division protein Gid	5
K04096	DNA processing protein	5
K05592	ATP-dependent RNA helicase DeaD [EC:3.6.4.13]	5
K06153	undecaprenyl-diphosphatase [EC:3.6.1.27]	5
K07042	probable rRNA maturation factor	5
K07192	flotillin	5
K07391	magnesium chelatase family protein	5
K07407	alpha-galactosidase [EC:3.2.1.22]	5
K07458	DNA mismatch endonuclease, patch repair protein [EC:3.1.-.-]	5

Continued on next page

K07566	putative translation factor	5
K07668	two-component system, OmpR family, response regulator VicR	5
K07712	two-component system, NtrC family, nitrogen regulation response regulator GlnG	5
K07776	two-component system, OmpR family, response regulator RegX3	5
K08309	soluble lytic murein transglycosylase [EC:3.2.1.-]	5
K08641	D-alanyl-D-alanine dipeptidase [EC:3.4.13.-]	5
K08998	hypothetical protein	5
K09747	hypothetical protein	5
K09808	lipoprotein-releasing system permease protein	5
K09810	lipoprotein-releasing system ATP-binding protein [EC:3.6.3.-]	5
K10026	queuosine biosynthesis protein QueE	5
K11717	cysteine desulfurase / selenocysteine lyase [EC:2.8.1.7 4.4.1.16]	5
K00172	pyruvate ferredoxin oxidoreductase, gamma subunit [EC:1.2.7.1]	6
K00319	methylenetetrahydromethanopterin dehydrogenase [EC:1.5.99.9]	6
K00320	coenzyme F420-dependent N5,N10-methenyltetrahydromethanopterin reductase [EC:1.5.99.11]	6
K00583	tetrahydromethanopterin S-methyltransferase subunit G [EC:2.1.1.86]	6
K01516	nucleoside-triphosphatase [EC:3.6.1.15]	6
K01854	UDP-galactopyranose mutase [EC:5.4.99.9]	6
K01933	phosphoribosylformylglycinamidine cyclo-ligase [EC:6.3.3.1]	6
K01985	5S ribosomal RNA	6
K02120	V-type H+-transporting ATPase subunit D [EC:3.6.3.14]	6
K02224	cobyrinic acid a,c-diamide synthase [EC:6.3.5.9 6.3.1.-]	6
K02427	ribosomal RNA large subunit methyltransferase E [EC:2.1.1.-]	6
K02440	glycerol uptake facilitator protein	6
K02866	large subunit ribosomal protein L10e	6
K02869	large subunit ribosomal protein L12	6
K02875	large subunit ribosomal protein L14e	6
K02883	large subunit ribosomal protein L18e	6
K02896	large subunit ribosomal protein L24e	6
K02904	large subunit ribosomal protein L29	6
K02910	large subunit ribosomal protein L31e	6
K02921	large subunit ribosomal protein L37Ae	6
K02922	large subunit ribosomal protein L37e	6
K02924	large subunit ribosomal protein L39e	6
K02927	large subunit ribosomal protein L40e	6
K02929	large subunit ribosomal protein L44e	6
K02936	large subunit ribosomal protein L7Ae	6
K02948	small subunit ribosomal protein S11	6
K02950	small subunit ribosomal protein S12	6
K02952	small subunit ribosomal protein S13	6
K02962	small subunit ribosomal protein S17e	6

Continued on next page

K02966	small subunit ribosomal protein S19e	6
K02977	small subunit ribosomal protein S27Ae	6
K02978	small subunit ribosomal protein S27e	6
K02991	small subunit ribosomal protein S6e	6
K02994	small subunit ribosomal protein S8	6
K02995	small subunit ribosomal protein S8e	6
K03056	DNA-directed RNA polymerase subunit L [EC:2.7.7.6]	6
K03058	DNA-directed RNA polymerase subunit N [EC:2.7.7.6]	6
K03231	elongation factor EF-1 alpha subunit [EC:3.6.5.3]	6
K03238	translation initiation factor eIF-2 beta subunit	6
K03263	translation initiation factor eIF-5A	6
K03389	heterodisulfide reductase subunit B [EC:1.8.98.1]	6
K03538	ribonuclease P protein subunit POP4 [EC:3.1.26.5]	6
K03540	ribonuclease P protein subunit RPR2 [EC:3.1.26.5]	6
K03768	peptidyl-prolyl cis-trans isomerase B (cyclophilin B) [EC:5.2.1.8]	6
K03775	FKBP-type peptidyl-prolyl cis-trans isomerase SlyD [EC:5.2.1.8]	6
K04796	small nuclear ribonucleoprotein	6
K04797	prefoldin alpha subunit	6
K04802	proliferating cell nuclear antigen	6
K06891	ATP-dependent Clp protease adaptor protein ClpS	6
K06941	ribosomal RNA large subunit methyltransferase N [EC:2.1.1.-]	6
K06961	ribosomal RNA assembly protein	6
K07107	acyl-CoA thioester hydrolase [EC:3.1.2.-]	6
K07580	hypothetical protein	6
K07582	hypothetical protein	6
K07721	ArsR family transcriptional regulator	6
K09007	hypothetical protein	6
K09735	hypothetical protein	6
K09858	SEC-C motif domain protein	6
K11176	IMP cyclohydrolase [EC:3.5.4.10]	6
K14124	energy-converting hydrogenase B subunit O	6
K14127	F420-non-reducing hydrogenase iron-sulfur subunit D [EC:1.12.99.-]	6
K14230	tRNA Met	6
K00134	glyceraldehyde 3-phosphate dehydrogenase [EC:1.2.1.12]	7
K00174	2-oxoglutarate ferredoxin oxidoreductase subunit alpha [EC:1.2.7.3]	7
K00175	2-oxoglutarate ferredoxin oxidoreductase subunit beta [EC:1.2.7.3]	7
K00177	2-oxoglutarate ferredoxin oxidoreductase subunit gamma [EC:1.2.7.3]	7
K00239	succinate dehydrogenase flavoprotein subunit [EC:1.3.99.1]	7
K00240	succinate dehydrogenase iron-sulfur protein [EC:1.3.99.1]	7
K00330	NADH dehydrogenase I subunit A [EC:1.6.5.3]	7
K00338	NADH dehydrogenase I subunit I [EC:1.6.5.3]	7
K00341	NADH dehydrogenase I subunit L [EC:1.6.5.3]	7
K00347	Na+-transporting NADH:ubiquinone oxidoreductase subunit B [EC:1.6.5.-]	7

Continued on next page

Table S2 – Continued from previous page

K00349	Na+-transporting NADH:ubiquinone oxidoreductase subunit D [EC:1.6.5.-]	7
K00350	Na+-transporting NADH:ubiquinone oxidoreductase subunit E [EC:1.6.5.-]	7
K00351	Na+-transporting NADH:ubiquinone oxidoreductase subunit F [EC:1.6.5.-]	7
K00666	fatty-acyl-CoA synthase [EC:6.2.1.-]	7
K00831	phosphoserine aminotransferase [EC:2.6.1.52]	7
K00874	2-dehydro-3-deoxygluconokinase [EC:2.7.1.45]	7
K00895	pyrophosphate--fructose-6-phosphate 1-phosphotransferase [EC:2.7.1.90]	7
K00927	phosphoglycerate kinase [EC:2.7.2.3]	7
K00962	polyribonucleotide nucleotidyltransferase [EC:2.7.7.8]	7
K01425	glutaminase [EC:3.5.1.2]	7
K01625	2-dehydro-3-deoxyphosphogluconate aldolase / 4-hydroxy-2-oxoglutarate aldolase [EC:4.1.2.14 4.1.3.16]	7
K01676	fumarate hydratase, class I [EC:4.2.1.2]	7
K01803	triosephosphate isomerase (TIM) [EC:5.3.1.1]	7
K01812	glucuronate isomerase [EC:5.3.1.12]	7
K01847	methylmalonyl-CoA mutase [EC:5.4.99.2]	7
K01868	threonyl-tRNA synthetase [EC:6.1.1.3]	7
K01880	glycyl-tRNA synthetase [EC:6.1.1.14]	7
K02355	elongation factor EF-G [EC:3.6.5.3]	7
K02357	elongation factor EF-Ts	7
K02876	large subunit ribosomal protein L15	7
K02926	large subunit ribosomal protein L4	7
K02933	large subunit ribosomal protein L6	7
K02945	small subunit ribosomal protein S1	7
K02959	small subunit ribosomal protein S16	7
K02982	small subunit ribosomal protein S3	7
K03305	proton-dependent oligopeptide transporter, POT family	7
K03521	electron transfer flavoprotein beta subunit	7
K03522	electron transfer flavoprotein alpha subunit	7
K03561	biopolymer transport protein ExbB	7
K06950	uncharacterized protein	7
K13378	NADH dehydrogenase I subunit C/D [EC:1.6.5.3]	7
K00013	histidinol dehydrogenase [EC:1.1.1.23]	8
K00287	dihydrofolate reductase [EC:1.5.1.3]	8
K00604	methionyl-tRNA formyltransferase [EC:2.1.2.9]	8
K00791	tRNA dimethylallyltransferase [EC:2.5.1.75]	8
K00845	glucokinase [EC:2.7.1.2]	8
K00942	guanylate kinase [EC:2.7.4.8]	8
K00951	GTP pyrophosphokinase [EC:2.7.6.5]	8
K01591	orotidine-5 -phosphate decarboxylase [EC:4.1.1.23]	8
K01627	2-dehydro-3-deoxyphosphooctonate aldolase (KDO 8-P synthase) [EC:2.5.1.55]	8
K01921	D-alanine-D-alanine ligase [EC:6.3.2.4]	8
K01929	UDP-N-acetylmuramoylalanyl-D-glutamyl-2,6-diaminopimelate--D-alanyl-D-alanine ligase [EC:6.3.2.10]	8
K01972	DNA ligase (NAD+) [EC:6.5.1.2]	8
K02493	methyltransferase [EC:2.1.1.-]	8

Continued on next page

K02503	Hit-like protein involved in cell-cycle regulation	8
K02563	UDP-N-acetylglucosamine--N-acetylmuramyl-(pentapeptide) pyrophosphoryl-undecaprenol N-acetylglucosamine transferase [EC:2.4.1.227]	8
K02838	ribosome recycling factor	8
K03527	4-hydroxy-3-methylbut-2-enyl diphosphate reductase [EC:1.17.1.2]	8
K03555	DNA mismatch repair protein MutS	8
K03572	DNA mismatch repair protein MutL	8
K03575	A/G-specific adenine glycosylase [EC:3.2.2.-]	8
K03584	DNA repair protein RecO (recombination protein O)	8
K03624	transcription elongation factor GreA	8
K04068	anaerobic ribonucleoside-triphosphate reductase activating protein [EC:1.97.1.4]	8
K05366	penicillin-binding protein 1A [EC:2.4.1.- 3.4.-.-]	8
K05515	penicillin-binding protein 2	8
K06215	pyridoxine biosynthesis protein [EC:4.-.-.-]	8
K06949	ribosome biogenesis GTPase [EC:3.6.1.-]	8
K07058	membrane protein	8
K10039	putative glutamine transport system substrate-binding protein	8
K11175	phosphoribosylglycinamide formyltransferase 1 [EC:2.1.2.2]	8
K12251	N-carbamoylputrescine amidase [EC:3.5.1.53]	8
K12573	ribonuclease R [EC:3.1.-.-]	8
K13038	phosphopantothenoylcysteine decarboxylase / phosphopantothenate--cysteine ligase [EC:4.1.1.36 6.3.2.5]	8
K13747	carboxynorspermidine decarboxylase [EC:4.1.1.-]	8
K13789	geranylgeranyl diphosphate synthase, type II [EC:2.5.1.1 2.5.1.10 2.5.1.29]	8
K00040	fructuronate reductase [EC:1.1.1.57]	9
K00283	glycine dehydrogenase subunit 2 [EC:1.4.4.2]	9
K00425	cytochrome bd-I oxidase subunit I [EC:1.10.3.-]	9
K00677	UDP-N-acetylglucosamine acyltransferase [EC:2.3.1.129]	9
K00765	ATP phosphoribosyltransferase [EC:2.4.2.17]	9
K00849	galactokinase [EC:2.7.1.6]	9
K01089	imidazoleglycerol-phosphate dehydratase / histidinol-phosphatase [EC:4.2.1.19 3.1.3.15]	9
K01195	beta-glucuronidase [EC:3.2.1.31]	9
K01200	pullulanase [EC:3.2.1.41]	9
K01284	peptidyl-dipeptidase Dcp [EC:3.4.15.5]	9
K01661	naphthoate synthase [EC:4.1.3.36]	9
K01808	ribose 5-phosphate isomerase B [EC:5.3.1.6]	9
K01811	putative family 31 glucosidase	9
K01834	phosphoglycerate mutase [EC:5.4.2.1]	9
K01867	tryptophanyl-tRNA synthetase [EC:6.1.1.2]	9
K01874	methionyl-tRNA synthetase [EC:6.1.1.10]	9
K01889	phenylalanyl-tRNA synthetase alpha chain [EC:6.1.1.20]	9
K02113	F-type H+-transporting ATPase subunit delta [EC:3.6.3.14]	9
K02548	1,4-dihydroxy-2-naphthoate octaprenyltransferase [EC:2.5.1.74]	9

Continued on next page

Table S2 – Continued from previous page

K02564	glucosamine-6-phosphate deaminase [EC:3.5.99.6]	9
K02879	large subunit ribosomal protein L17	9
K02892	large subunit ribosomal protein L23	9
K02911	large subunit ribosomal protein L32	9
K02939	large subunit ribosomal protein L9	9
K03088	RNA polymerase sigma-70 factor, ECF subfamily	9
K03217	preprotein translocase subunit YidC	9
K03588	cell division protein FtsW	9
K05349	beta-glucosidase [EC:3.2.1.21]	9
K07214	enterochelin esterase and related enzymes	9
K07386	putative endopeptidase [EC:3.4.24.-]	9
K07588	LAO/AO transport system kinase [EC:2.7.-.-]	9
K08138	MFS transporter, SP family, xylose:H+ symportor	9
K09013	Fe-S cluster assembly ATP-binding protein	9
K00024	malate dehydrogenase [EC:1.1.1.37]	10
K00036	glucose-6-phosphate 1-dehydrogenase [EC:1.1.1.49]	10
K00290	saccharopine dehydrogenase (NAD+, L-lysine forming) [EC:1.5.1.7]	10
K00348	Na+-transporting NADH:ubiquinone oxidoreductase subunit C [EC:1.6.5.-]	10
K00645	[acyl-carrier-protein] S-malonyltransferase [EC:2.3.1.39]	10
K00760	hypoxanthine phosphoribosyltransferase [EC:2.4.2.8]	10
K00766	anthranilate phosphoribosyltransferase [EC:2.4.2.18]	10
K01358	ATP-dependent Clp protease, protease subunit [EC:3.4.21.92]	10
K01872	alanyl-tRNA synthetase [EC:6.1.1.7]	10
K01919	glutamate--cysteine ligase [EC:6.3.2.2]	10
K01923	phosphoribosylaminoimidazole-succinocarboxamide synthase [EC:6.3.2.6]	10
K02342	DNA polymerase III subunit epsilon [EC:2.7.7.7]	10
K02532	MFS transporter, OHS family, lactose permease	10
K02600	N utilization substance protein A	10
K02796	PTS system, mannose-specific IID component	10
K03148	adenylyltransferase [EC:2.7.7.-]	10
K03289	MFS transporter, NHS family, nucleoside permease	10
K03293	amino acid transporter, AAT family	10
K03654	ATP-dependent DNA helicase RecQ [EC:3.6.4.12]	10
K07568	S-adenosylmethionine:tRNA ribosyltransferase-isomerase [EC:5.-.-.-]	10
K08300	ribonuclease E [EC:3.1.26.12]	10
K09014	Fe-S cluster assembly protein SufB	10
K09118	hypothetical protein	10
K09458	3-oxoacyl-[acyl-carrier-protein] synthase II [EC:2.3.1.179]	10
K09903	uridylate kinase [EC:2.7.4.22]	10
K11263	acetyl-/propionyl-CoA carboxylase, biotin carboxylase, biotin carboxyl carrier protein [EC:6.3.4.14]	10
K11533	fatty acid synthase, bacteria type [EC:2.3.1.-]	10
K14623	DNA-damage-inducible protein D	10
K00864	glycerol kinase [EC:2.7.1.30]	11
K01223	6-phospho-beta-glucosidase [EC:3.2.1.86]	11
K01507	inorganic pyrophosphatase [EC:3.6.1.1]	11

Continued on next page

K01679	fumarate hydratase, class II [EC:4.2.1.2]	11
K01712	urocanate hydratase [EC:4.2.1.49]	11
K01752	L-serine dehydratase [EC:4.3.1.17]	11
K01937	CTP synthase [EC:6.3.4.2]	11
K01963	acetyl-CoA carboxylase carboxyl transferase subunit beta [EC:6.4.1.2]	11
K01996	branched-chain amino acid transport system ATP-binding protein	11
K02010	iron(III) transport system ATP-binding protein [EC:3.6.3.30]	11
K02028	polar amino acid transport system ATP-binding protein [EC:3.6.3.21]	11
K02112	F-type H+-transporting ATPase subunit beta [EC:3.6.3.14]	11
K03086	RNA polymerase primary sigma factor	11
K03544	ATP-dependent Clp protease ATP-binding subunit ClpX	11
K03553	recombination protein RecA	11
K03596	GTP-binding protein LepA	11
K03628	transcription termination factor Rho	11
K03695	ATP-dependent Clp protease ATP-binding subunit ClpB	11
K03798	cell division protease FtsH [EC:3.4.24.-]	11
K03856	3-deoxy-7-phosphoheptulonate synthase [EC:2.5.1.54]	11
K09157	hypothetical protein	11
K09691	lipopolysaccharide transport system ATP-binding protein	11
K10008	glutamate transport system ATP-binding protein [EC:3.6.3.-]	11
K10112	maltose/maltodextrin transport system ATP-binding protein	11
K00226	dihydroorotate oxidase [EC:1.3.3.1]	12
K00928	aspartate kinase [EC:2.7.2.4]	12
K01190	beta-galactosidase [EC:3.2.1.23]	12
K01738	cysteine synthase A [EC:2.5.1.47]	12
K01770	2-C-methyl-D-erythritol 2,4-cyclodiphosphate synthase [EC:4.6.1.12]	12
K01918	pantoate--beta-alanine ligase [EC:6.3.2.1]	12
K02426	cysteine desulfuration protein SufE	12
K02428	nucleoside-triphosphate pyrophosphatase [EC:3.6.1.19]	12
K02523	octaprenyl diphosphate synthase [EC:2.5.1.-]	12
K02536	UDP-3-O-[3-hydroxymyristoyl] glucosamine N-acyltransferase [EC:2.3.1.-]	12
K02834	ribosome-binding factor A	12
K03545	trigger factor	12
K03587	cell division protein FtsI (penicillin-binding protein 3) [EC:2.4.1.129]	12
K03800	lipoate-protein ligase A [EC:2.7.7.63]	12
K03925	MraZ protein	12
K03977	GTP-binding protein	12
K05807	putative lipoprotein	12
K07560	D-tyrosyl-tRNA(Tyr) deacylase [EC:3.1.-.-]	12
K12340	outer membrane channel protein TolC	12
K00278	L-aspartate oxidase [EC:1.4.3.16]	13
K00324	NAD(P) transhydrogenase subunit alpha [EC:1.6.1.2]	13
K00325	NAD(P) transhydrogenase subunit beta [EC:1.6.1.2]	13

Continued on next page

Table S2 – Continued from previous page

K00656	formate C-acetyltransferase [EC:2.3.1.54]	13
K00873	pyruvate kinase [EC:2.7.1.40]	13
K01090	protein phosphatase [EC:3.1.3.16]	13
K01595	phosphoenolpyruvate carboxylase [EC:4.1.1.31]	13
K01607	4-carboxymuconolactone decarboxylase [EC:4.1.1.44]	13
K01784	UDP-glucose 4-epimerase [EC:5.1.3.2]	13
K01958	pyruvate carboxylase [EC:6.4.1.1]	13
K03294	basic amino acid/polyamine antiporter, APA family	13
K03549	KUP system potassium uptake protein	13
K03615	electron transport complex protein RnfC	13
K07459	putative ATP-dependent endonuclease of the OLD family	13
K08659	dipeptidase [EC:3.4.-.-]	13
K11104	melibiose permease	13
K00626	acetyl-CoA C-acetyltransferase [EC:2.3.1.9]	14
K00963	UTP--glucose-1-phosphate uridylyltransferase [EC:2.7.7.9]	14
K01778	diaminopimelate epimerase [EC:5.1.1.7]	14
K01902	succinyl-CoA synthetase alpha subunit [EC:6.2.1.5]	14
K02020	molybdate transport system substrate-binding protein	14
K02033	peptide/nickel transport system permease protein	14
K02189	cobalamin biosynthesis protein CbiG	14
K02232	adenosylcobyric acid synthase [EC:6.3.5.10]	14
K03072	preprotein translocase subunit SecD	14
K03147	thiamine biosynthesis protein ThiC	14
K03639	molybdenum cofactor biosynthesis protein	14
K03699	putative hemolysin	14
K05934	precorrin-3B C17-methyltransferase [EC:2.1.1.131]	14
K00854	xylulokinase [EC:2.7.1.17]	15
K02518	translation initiation factor IF-1	15
K02887	large subunit ribosomal protein L20	15
K02888	large subunit ribosomal protein L21	15
K02909	large subunit ribosomal protein L31	15
K02913	large subunit ribosomal protein L33	15
K02968	small subunit ribosomal protein S20	15
K03502	DNA polymerase V	15
K03517	quinolinate synthase [EC:2.5.1.72]	15
K03530	DNA-binding protein HU-beta	15
K03617	electron transport complex protein RnfA	15
K03719	Lrp/AsnC family transcriptional regulator, leucine-responsive regulatory protein	15
K03803	sigma-E factor negative regulatory protein RseC	15
K05808	putative sigma-54 modulation protein	15
K00346	Na+-transporting NADH:ubiquinone oxidoreductase subunit A [EC:1.6.5.-]	16
K00812	aspartate aminotransferase [EC:2.6.1.1]	16
K01624	fructose-bisphosphate aldolase, class II [EC:4.1.2.13]	16
K01840	phosphomannomutase [EC:5.4.2.8]	16
K03595	GTP-binding protein Era	16
K06142	outer membrane protein	16
K06178	ribosomal large subunit pseudouridine synthase B [EC:5.4.99.12]	16
K07792	anaerobic C4-dicarboxylate transporter DcuB	16

Continued on next page

K12524	bifunctional aspartokinase / homoserine dehydrogenase 1 [EC:2.7.2.4 1.1.1.3]	16
K13043	N-succinyl-L-ornithine transcarbamylase [EC:2.1.3.11]	16
K00074	3-hydroxybutyryl-CoA dehydrogenase [EC:1.1.1.157]	17
K00789	S-adenosylmethionine synthetase [EC:2.5.1.6]	17
K03413	two-component system, chemotaxis family, response regulator CheY	17
K03667	ATP-dependent HslUV protease ATP-binding subunit HslU	17
K03701	excinuclease ABC subunit A	17
K06020	sulfate-transporting ATPase [EC:3.6.3.25]	17
K06217	phosphate starvation-inducible protein PhoH and related proteins	17
K09972	general L-amino acid transport system ATP-binding protein [EC:3.6.3.-]	17
K10041	putative glutamine transport system ATP-binding protein [EC:3.6.3.-]	17
K14226	tRNA His	17
K00721	dolichol-phosphate mannosyltransferase [EC:2.4.1.83]	18
K00748	lipid-A-disaccharide synthase [EC:2.4.1.182]	18
K01585	arginine decarboxylase [EC:4.1.1.19]	18
K03978	GTP-binding protein	18
K04564	superoxide dismutase, Fe-Mn family [EC:1.15.1.1]	18
K04773	protease IV [EC:3.4.21.-]	18
K06861	lipopolysaccharide export system ATP-binding protein [EC:3.6.3.-]	18
K07259	D-alanyl-D-alanine carboxypeptidase / D-alanyl-D-alanine-endopeptidase (penicillin-binding protein 4) [EC:3.4.16.4 3.4.99.-]	18
K07315	sigma-B regulation protein RsbU (phosphoserine phosphatase)	18
K08307	membrane-bound lytic murein transglycosylase D [EC:3.2.1.-]	18
K00033	6-phosphogluconate dehydrogenase [EC:1.1.1.44]	19
K00091	dihydroflavonol-4-reductase [EC:1.1.1.219]	19
K00527	ribonucleoside-triphosphate reductase [EC:1.17.4.2]	19
K00639	glycine C-acetyltransferase [EC:2.3.1.29]	19
K00657	diamine N-acetyltransferase [EC:2.3.1.57]	19
K02052	putative spermidine/putrescine transport system ATP-binding protein	19
K02056	simple sugar transport system ATP-binding protein [EC:3.6.3.17]	19
K02117	V-type H+-transporting ATPase subunit A [EC:3.6.3.14]	19
K03767	peptidyl-prolyl cis-trans isomerase A (cyclophilin A) [EC:5.2.1.8]	19
K10548	putative multiple sugar transport system ATP-binding protein	19
K00031	isocitrate dehydrogenase [EC:1.1.1.42]	20
K00528	ferredoxin--NADP+ reductase [EC:1.18.1.2]	20
K00611	ornithine carbamoyltransferase [EC:2.1.3.3]	20
K00948	ribose-phosphate pyrophosphokinase [EC:2.7.6.1]	20
K02032	peptide/nickel transport system ATP-binding protein	20
K02034	peptide/nickel transport system permease protein	20

Continued on next page

K02825	pyrimidine operon attenuation protein / uracil phosphoribosyltransferase [EC:2.4.2.9]	20
K04072	acetaldehyde dehydrogenase / alcohol dehydrogenase [EC:1.2.1.10 1.1.1.1]	20
K06959	uncharacterized protein	20
K09812	cell division transport system ATP-binding protein	20
K00526	ribonucleoside-diphosphate reductase beta chain [EC:1.17.4.1]	21
K00925	acetate kinase [EC:2.7.2.1]	21
K01577	oxalyl-CoA decarboxylase [EC:4.1.1.8]	21
K01632	fructose-6-phosphate phosphoketolase [EC:4.1.2.22]	21
K01835	phosphoglucomutase [EC:5.4.2.2]	21
K01885	glutamyl-tRNA synthetase [EC:6.1.1.17]	21
K03585	membrane fusion protein	21
K07749	formyl-CoA transferase [EC:2.8.3.16]	21
K10005	glutamate transport system substrate-binding protein	21
K10007	glutamate transport system permease protein	21
K00179	indolepyruvate ferredoxin oxidoreductase, alpha subunit [EC:1.2.7.8]	22
K00231	protoporphyrinogen oxidase [EC:1.3.3.4]	22
K00382	dihydrolipoamide dehydrogenase [EC:1.8.1.4]	22
K00634	phosphate butyryltransferase [EC:2.3.1.19]	22
K01235	alpha-glucuronidase [EC:3.2.1.139]	22
K01733	threonine synthase [EC:4.2.3.1]	22
K02051	sulfonate/nitrate/taurine transport system substrate-binding protein	22
K07170	GAF domain-containing protein	22
K09797	hypothetical protein	22
K09955	hypothetical protein	22
K01428	urease subunit alpha [EC:3.5.1.5]	23
K01999	branched-chain amino acid transport system substrate-binding protein	23
K02282	pilus assembly protein CpaE	23
K03168	DNA topoisomerase I [EC:5.99.1.2]	23
K03758	arginine:ornithine antiporter	23
K03892	ArsR family transcriptional regulator	23
K07481	transposase, IS5 family	23
K07483	transposase	23
K12510	tight adherence protein B	23
K00342	NADH dehydrogenase I subunit M [EC:1.6.5.3]	24
K00946	thiamine-monophosphate kinase [EC:2.7.4.16]	24
K01883	cysteinyl-tRNA synthetase [EC:6.1.1.16]	24
K02621	topoisomerase IV subunit A [EC:5.99.1.-]	24
K03281	chloride channel protein, CIC family	24
K03590	cell division protein FtsA	24
K07277	outer membrane protein	24
K08289	phosphoribosylglycinamide formyltransferase 2 [EC:2.1.2.2]	24
K00052	3-isopropylmalate dehydrogenase [EC:1.1.1.85]	25
K00800	3-phosphoshikimate 1-carboxyvinyltransferase [EC:2.5.1.19]	25

Continued on next page

K00820	glucosamine--fructose-6-phosphate aminotransferase (isomerizing) [EC:2.6.1.16]	25
K01338	ATP-dependent Lon protease [EC:3.4.21.53]	25
K01653	acetolactate synthase I/III small subunit [EC:2.2.1.6]	25
K01703	3-isopropylmalate/(R)-2-methylmalate dehydratase large subunit [EC:4.2.1.33 4.2.1.35]	25
K01714	dihydrodipicolinate synthase [EC:4.2.1.52]	25
K01756	adenylosuccinate lyase [EC:4.3.2.2]	25
K02283	pilus assembly protein CpaF	25
K00578	tetrahydromethanopterin S-methyltransferase subunit B [EC:2.1.1.86]	26
K00978	glucose-1-phosphate cytidylyltransferase [EC:2.7.7.33]	26
K03330	glutamyl-tRNA(Gln) amidotransferase subunit E [EC:6.3.5.7]	26
K07491	putative transposase	26
K11646	dehydroquinate synthase II [EC:1.4.1.-]	26
K14081	methanol corrinoid protein	26
K14218	tRNA Ala	26
K14227	tRNA Ile	26
K14235	tRNA Trp	26
K00609	aspartate carbamoyltransferase catalytic subunit [EC:2.1.3.2]	27
K01448	N-acetylmuramoyl-L-alanine amidase [EC:3.5.1.28]	27
K02108	F-type H+-transporting ATPase subunit a [EC:3.6.3.14]	27
K02188	cobalamin biosynthesis protein CbiD	27
K03499	trk system potassium uptake protein TrkA	27
K07173	S-ribosylhomocysteine lyase [EC:4.4.1.21]	27
K07727	putative transcriptional regulator	27
K02110	F-type H+-transporting ATPase subunit c [EC:3.6.3.14]	28
K02190	sirohydrochlorin cobaltochelatase [EC:4.99.1.3]	28
K02348	ElaA protein	28
K02884	large subunit ribosomal protein L19	28
K02899	large subunit ribosomal protein L27	28
K02914	large subunit ribosomal protein L34	28
K02954	small subunit ribosomal protein S14	28
K05516	curved DNA-binding protein	28
K01188	beta-glucosidase [EC:3.2.1.21]	29
K01259	proline iminopeptidase [EC:3.4.11.5]	29
K01804	L-arabinose isomerase [EC:5.3.1.4]	29
K01870	isoleucyl-tRNA synthetase [EC:6.1.1.5]	29
K03106	signal recognition particle subunit SRP54	29
K03307	solute:Na+ symporter, SSS family	29
K03503	DNA polymerase V [EC:3.4.21.-]	29
K08191	MFS transporter, ACS family, hexuronate transporter	29
K00931	glutamate 5-kinase [EC:2.7.2.11]	30
K01209	alpha-N-arabinofuranosidase [EC:3.2.1.55]	30
K01875	seryl-tRNA synthetase [EC:6.1.1.11]	30
K01887	arginyl-tRNA synthetase [EC:6.1.1.19]	30
K02314	replicative DNA helicase [EC:3.6.4.12]	30
K02528	16S rRNA (adenine1518-N6/adenine1519-N6)-dimethyltransferase [EC:2.1.1.182]	30

Continued on next page

Table S2 – Continued from previous page

K02837	peptide chain release factor RF-3	30
K03526	(E)-4-hydroxy-3-methylbut-2-enyl-diphosphate synthase [EC:1.17.7.1]	30
K00241	succinate dehydrogenase cytochrome b-556 subunit	31
K02863	large subunit ribosomal protein L1	31
K02867	large subunit ribosomal protein L11	31
K02906	large subunit ribosomal protein L3	31
K02931	large subunit ribosomal protein L5	31
K02961	small subunit ribosomal protein S17	31
K13993	HSP20 family protein	31
K14222	tRNA Cys	31
K00335	NADH dehydrogenase I subunit F [EC:1.6.5.3]	32
K00973	glucose-1-phosphate thymidylyltransferase [EC:2.7.7.24]	32
K01251	adenosylhomocysteinase [EC:3.3.1.1]	32
K01596	phosphoenolpyruvate carboxykinase (GTP) [EC:4.1.1.32]	32
K01696	tryptophan synthase beta chain [EC:4.2.1.20]	32
K01704	3-isopropylmalate/(R)-2-methylmalate dehydratase small subunit [EC:4.2.1.33 4.2.1.35]	32
K02031	peptide/nickel transport system ATP-binding protein	32
K02049	sulfonate/nitrate/taurine transport system ATP-binding protein	32
K02864	large subunit ribosomal protein L10	33
K02871	large subunit ribosomal protein L13	33
K02890	large subunit ribosomal protein L22	33
K02895	large subunit ribosomal protein L24	33
K02956	small subunit ribosomal protein S15	33
K02992	small subunit ribosomal protein S7	33
K02996	small subunit ribosomal protein S9	33
K01433	formyltetrahydrofolate deformylase [EC:3.5.1.10]	34
K01960	pyruvate carboxylase subunit B [EC:6.4.1.1]	34
K03303	lactate transporter, LctP family	34
K03832	periplasmic protein TonB	34
K05801	DnaJ like chaperone protein	34
K09011	D-citramalate synthase [EC:2.3.1.182]	34
K11749	regulator of sigma E protease [EC:3.4.24.-]	34
K00059	3-oxoacyl-[acyl-carrier protein] reductase [EC:1.1.1.100]	35
K00265	glutamate synthase (NADPH/NADH) large chain [EC:1.4.1.13 1.4.1.14]	35
K00266	glutamate synthase (NADPH/NADH) small chain [EC:1.4.1.13 1.4.1.14]	35
K00615	transketolase [EC:2.2.1.1]	35
K01952	phosphoribosylformylglycinamidine synthase [EC:6.3.5.3]	35
K04567	lysyl-tRNA synthetase, class II [EC:6.1.1.6]	35
K04761	LysR family transcriptional regulator, hydrogen peroxide-inducible genes activator	35
K01006	pyruvate,orthophosphate dikinase [EC:2.7.9.1]	36
K01610	phosphoenolpyruvate carboxykinase (ATP) [EC:4.1.1.49]	36
K01629	rhamnulose-1-phosphate aldolase [EC:4.1.2.19]	36
K02111	F-type H+-transporting ATPase subunit alpha [EC:3.6.3.14]	36
K03737	putative pyruvate-flavodoxin oxidoreductase [EC:1.2.7.-]	36
K04043	molecular chaperone DnaK	36

Continued on next page

Table S2 – Continued from previous page

K02520	translation initiation factor IF-3	37
K02527	3-deoxy-D-manno-octulosonic-acid transferase [EC:2.-.-.-]	37
K02622	topoisomerase IV subunit B [EC:5.99.1.-]	37
K04518	prephenate dehydratase [EC:4.2.1.51]	37
K07148	uncharacterized protein	37
K00058	D-3-phosphoglycerate dehydrogenase [EC:1.1.1.95]	38
K00262	glutamate dehydrogenase (NADP+) [EC:1.4.1.4]	38
K00600	glycine hydroxymethyltransferase [EC:2.1.2.1]	38
K01895	acetyl-CoA synthetase [EC:6.2.1.1]	38
K01912	phenylacetate-CoA ligase [EC:6.2.1.30]	38
K03569	rod shape-determining protein MreB and related proteins	38
K01586	diaminopimelate decarboxylase [EC:4.1.1.20]	39
K01749	hydroxymethylbilane synthase [EC:2.5.1.61]	39
K02035	peptide/nickel transport system substrate-binding protein	39
K02500	cyclase [EC:4.1.3.-]	39
K03177	tRNA pseudouridine synthase B [EC:5.4.99.12]	39
K07025	putative hydrolase of the HAD superfamily	39
K01940	argininosuccinate synthase [EC:6.3.4.5]	40
K02874	large subunit ribosomal protein L14	40
K02907	large subunit ribosomal protein L30	40
K02967	small subunit ribosomal protein S2	40
K02986	small subunit ribosomal protein S4	40
K02988	small subunit ribosomal protein S5	40
K02027	multiple sugar transport system substrate-binding protein	41
K02039	phosphate transport system protein	41
K02433	aspartyl-tRNA(Asn)/glutamyl-tRNA (Gln) amidotransferase subunit A [EC:6.3.5.6 6.3.5.7]	41
K03407	two-component system, chemotaxis family, sensor kinase CheA [EC:2.7.13.3]	41
K03724	ATP-dependent helicase Lhr and Lhr-like helicase [EC:3.6.4.-]	41
K04759	ferrous iron transport protein B	41
K01212	levanase [EC:3.2.1.65]	42
K02601	transcriptional antiterminator NusG	42
K02614	phenylacetic acid degradation protein	42
K05993	isochorismatase [EC:3.3.2.1]	42
K00705	4-alpha-glucanotransferase [EC:2.4.1.25]	43
K01491	methylenetetrahydrofolate dehydrogenase (NADP+) / methenyltetrahydrofolate cyclohydrolase [EC:1.5.1.5 3.5.4.9]	43
K01649	2-isopropylmalate synthase [EC:2.3.3.13]	43
K02316	DNA primase [EC:2.7.7.-]	43
K03927	carboxylesterase type B [EC:3.1.1.1]	43
K00768	nicotinate-nucleotide--dimethylbenzimidazole phosphoribosyltransferase [EC:2.4.2.21]	44
K03427	type I restriction enzyme M protein [EC:2.1.1.72]	44
K07496	putative transposase	44
K11085	ATP-binding cassette, subfamily B, bacterial MsbA [EC:3.6.3.-]	44
K14237	tRNA Val	44
K08217	MFS transporter, DHA3 family, macrolide efflux protein	45

Continued on next page

Table S2 – Continued from previous page

K14223	tRNA Gln	45
K14228	tRNA Leu	45
K14233	tRNA Ser	45
K14234	tRNA Thr	45
K00761	uracil phosphoribosyltransferase [EC:2.4.2.9]	46
K01588	5-(carboxyamino)imidazole ribonucleotide mutase [EC:5.4.99.18]	46
K03306	inorganic phosphate transporter, PiT family	46
K04042	bifunctional UDP-N-acetylglucosamine pyrophosphorylase / Glucosamine-1-phosphate N-acetyltransferase [EC:2.7.7.23 2.3.1.157]	46
K14219	tRNA Arg	46
K00912	tetraacyldisaccharide 4 -kinase [EC:2.7.1.130]	47
K01265	methionyl aminopeptidase [EC:3.4.11.18]	47
K01775	alanine racemase [EC:5.1.1.1]	47
K08301	ribonuclease G [EC:3.1.26.-]	47
K09015	Fe-S cluster assembly protein SufD	47
K00625	phosphate acetyltransferase [EC:2.3.1.8]	48
K01686	mannonate dehydratase [EC:4.2.1.8]	48
K01881	prolyl-tRNA synthetase [EC:6.1.1.15]	48
K02469	DNA gyrase subunit A [EC:5.99.1.3]	48
K02519	translation initiation factor IF-2	48
K00847	fructokinase [EC:2.7.1.4]	49
K00991	2-C-methyl-D-erythritol 4-phosphate cytidylyltransferase [EC:2.7.7.60]	49
K01262	Xaa-Pro aminopeptidase [EC:3.4.11.9]	49
K01939	adenylosuccinate synthase [EC:6.3.4.4]	49
K03614	electron transport complex protein RnfD	49
K00053	ketol-acid reductoisomerase [EC:1.1.1.86]	50
K00133	aspartate-semialdehyde dehydrogenase [EC:1.2.1.11]	50
K00560	thymidylate synthase [EC:2.1.1.45]	50
K00826	branched-chain amino acid aminotransferase [EC:2.6.1.42]	50
K01869	leucyl-tRNA synthetase [EC:6.1.1.4]	50
K01715	3-hydroxybutyryl-CoA dehydratase [EC:4.2.1.55]	51
K01755	argininosuccinate lyase [EC:4.3.2.1]	51
K03074	preprotein translocase subunit SecF	51
K03823	phosphinothricin acetyltransferase [EC:2.3.1.183]	51
K00145	N-acetyl-gamma-glutamyl-phosphate/N-acetyl-gamma-aminoadipyl-phosphate reductase [EC:1.2.1.38 1.2.1.-]	52
K00764	amidophosphoribosyltransferase [EC:2.4.2.14]	52
K00865	glycerate kinase [EC:2.7.1.31]	52
K01740	O-acetylhomoserine (thiol)-lyase [EC:2.5.1.49]	52
K00700	1,4-alpha-glucan branching enzyme [EC:2.4.1.18]	53
K01991	polysaccharide export outer membrane protein	53
K03547	exonuclease SbcD	53
K03783	purine-nucleoside phosphorylase [EC:2.4.2.1]	53
K01270	aminoacylhistidine dipeptidase [EC:3.4.13.3]	54
K03040	DNA-directed RNA polymerase subunit alpha [EC:2.7.7.6]	54
K01890	phenylalanyl-tRNA synthetase beta chain [EC:6.1.1.20]	55
K01892	histidyl-tRNA synthetase [EC:6.1.1.21]	55
K03797	carboxyl-terminal processing protease [EC:3.4.21.102]	55

Continued on next page

Table S2 – Continued from previous page

K04079	molecular chaperone HtpG	55
K02078	acyl carrier protein	56
K02902	large subunit ribosomal protein L28	56
K02946	small subunit ribosomal protein S10	56
K02990	small subunit ribosomal protein S6	56
K01783	ribulose-phosphate 3-epimerase [EC:5.1.3.1]	57
K01925	UDP-N-acetylmuramoylalanine--D-glutamate ligase [EC:6.3.2.9]	57
K02356	elongation factor EF-P	57
K03284	metal ion transporter, MIT family	57
K02775	PTS system, galactitol-specific IIC component	58
K03076	preprotein translocase subunit SecY	58
K04078	chaperonin GroES	58
K07405	alpha-amylase [EC:3.2.1.1]	58
K00088	IMP dehydrogenase [EC:1.1.1.205]	59
K01938	formate--tetrahydrofolate ligase [EC:6.3.4.3]	59
K03702	excinuclease ABC subunit B	59
K13892	glutathione transport system ATP-binding protein	59
K02358	elongation factor EF-Tu [EC:3.6.5.3]	60
K03043	DNA-directed RNA polymerase subunit beta [EC:2.7.7.6]	60
K03046	DNA-directed RNA polymerase subunit beta [EC:2.7.7.6]	60
K00097	4-hydroxythreonine-4-phosphate dehydrogenase [EC:1.1.1.262]	61
K03814	monofunctional biosynthetic peptidoglycan transglycosylase [EC:2.4.1.-]	61
K00215	dihydrodipicolinate reductase [EC:1.3.1.26]	62
K00432	glutathione peroxidase [EC:1.11.1.9]	62
K00939	adenylate kinase [EC:2.7.4.3]	62
K01620	threonine aldolase [EC:4.1.2.5]	63
K02337	DNA polymerase III subunit alpha [EC:2.7.7.7]	63
K03100	signal peptidase I [EC:3.4.21.89]	63
K00057	glycerol-3-phosphate dehydrogenase (NAD(P)+) [EC:1.1.1.94]	64
K03340	diaminopimelate dehydrogenase [EC:1.4.1.16]	64
K09760	DNA recombination protein RmuC	64
K01609	indole-3-glycerol phosphate synthase [EC:4.1.1.48]	65
K01678	fumarate hydratase subunit beta [EC:4.2.1.2]	65
K01903	succinyl-CoA synthetase beta subunit [EC:6.2.1.5]	65
K01786	L-ribulose-5-phosphate 4-epimerase [EC:5.1.3.4]	66
K02970	small subunit ribosomal protein S21	66
K09748	hypothetical protein	66
K01815	4-deoxy-L-threo-5-hexosulose-uronate ketol-isomerase [EC:5.3.1.17]	67
K01858	myo-inositol-1-phosphate synthase [EC:5.5.1.4]	67
K01677	fumarate hydratase subunit alpha [EC:4.2.1.2]	68
K05936	precorrin-4 C11-methyltransferase [EC:2.1.1.133]	68
K00945	cytidylate kinase [EC:2.7.4.14]	69
K03110	fused signal recognition particle receptor	69
K00041	tagaturonate reductase [EC:1.1.1.58]	70
K12257	SecD/SecF fusion protein	70
K00929	butyrate kinase [EC:2.7.2.7]	71

Continued on next page

Table S2 – Continued from previous page

K06199	CrcB protein	71
K00339	NADH dehydrogenase I subunit J [EC:1.6.5.3]	72
K00605	aminomethyltransferase [EC:2.1.2.10]	72
K00180	indolepyruvate ferredoxin oxidoreductase, beta subunit [EC:1.2.7.8]	73
K01710	dTDP-glucose 4,6-dehydratase [EC:4.2.1.46]	73
K00340	NADH dehydrogenase I subunit K [EC:1.6.5.3]	74
K02881	large subunit ribosomal protein L18	74
K00651	homoserine O-succinyltransferase [EC:2.3.1.46]	75
