# Supplementary material for: Bovine Host Genetic Variation Influences Rumen Microbial Methane Production with Best Selection Criterion for Low Methane Emitting and Efficiently Feed Converting Hosts Based on Metagenomic Gene Abundance
Source: PLoS Genet. 2016 Feb 18;12(2):e1005846. doi: 10.1371/journal.pgen.1005846 (PMC4758630; doi:10.1371/journal.pgen.1005846)
Supplement: S3 Table — Partial least squares (PLS) estimates of effects of microbial genes characterised by the Kyoto Encyclopedia of Genes and Genomes (KEGG) database in an analysis where the PLS factors explained 97.0% of the variation of model effects and 81.7% of the variation in methane emissions (g/kg dry matter intake). (PDF) [file pgen.1005846.s008.pdf]

Table S3. Microbial genes associated with methane emissions. Partial least squares (PLS) estimates of effects of microbial genes characterised by the Kyoto Encyclopedia of Genes and Genomes (KEGG) database in an analysis where the PLS factors explained 97.0 % of the variation in model effects and 81.7 % of the variation in methane emissions (g/kg dry matter intake).

KEGG ID, diet	Description	Estimate	VIP	P-value	R^2
Diet CON	concentrate based diet	-0.19980	1.314	0.001	0.459
Diet FOR	forage based diet	0.19980	1.314	0.001	0.459
K00584	tetrahydromethanopterin S-methyltransferase subunit H [EC:2.1.1.86]	0.07480	1.044	0.020	0.833
K00200	formylmethanofuran dehydrogenase subunit A [EC:1.2.99.5]	0.05914	0.998	0.038	0.789
K00205	formylmethanofuran dehydrogenase subunit F [EC:1.2.99.5]	0.05092	0.992	0.062	0.747
K00672	formylmethanofuran--tetrahydromethanopterin N-formyltransferase [EC:2.3.1.101]	0.04563	0.988	0.046	0.773
K00203	formylmethanofuran dehydrogenase subunit D [EC:1.2.99.5]	0.04299	0.985	0.025	0.819
K00169	pyruvate ferredoxin oxidoreductase, alpha subunit [EC:1.2.7.1]	0.03657	0.982	0.032	0.802
K00201	formylmethanofuran dehydrogenase subunit B [EC:1.2.99.5]	0.05767	0.973	0.013	0.859
K13812	bifunctional enzyme Fae/Hps [EC:4.3.-.- 4.1.2.43]	0.02893	0.965	0.051	0.765
K00580	tetrahydromethanopterin S-methyltransferase subunit D [EC:2.1.1.86]	0.04906	0.961	0.071	0.735
K00170	pyruvate ferredoxin oxidoreductase, beta subunit [EC:1.2.7.1]	0.02785	0.957	0.070	0.737
K00577	tetrahydromethanopterin S-methyltransferase subunit A [EC:2.1.1.86]	0.01639	0.957	0.063	0.747
K00400	methyl coenzyme M reductase system, component A2	0.01240	0.951	0.063	0.747
K14128	F420-non-reducing hydrogenase subunit G [EC:1.12.99.-]	0.03552	0.948	0.013	0.861
K00441	coenzyme F420 hydrogenase beta subunit [EC:1.12.98.1]	-0.00658	0.947	0.086	0.717
K14123	energy-converting hydrogenase B subunit N	0.00742	0.940	0.089	0.714
K00399	methyl-coenzyme M reductase alpha subunit [EC:2.8.4.1]	0.04127	0.937	0.010	0.872
K01499	methenyltetrahydromethanopterin cyclohydrolase [EC:3.5.4.27]	0.02985	0.934	0.038	0.790
K00123	formate dehydrogenase, alpha subunit [EC:1.2.1.2]	0.02813	0.931	0.013	0.857
K00581	tetrahydromethanopterin S-methyltransferase subunit E [EC:2.1.1.86]	0.02232	0.931	0.017	0.844
K00125	formate dehydrogenase, beta subunit [EC:1.2.1.2]	0.00544	0.929	0.030	0.808

Two factors were significant in the partial least squares analysis. Without considering diet effects in the model, the microbial genes explained 97.1 % of the variation of model effects and 77.1 % of the variation in methane emissions. The PLS estimates were based on predictors and responses centered and scaled to have mean 0 and standard deviation 1. Variable Importance for Projection (VIP) statistic of Wold, which summarizes the contribution of a variable maker to the model were used to identify the significant microbial genes associated with methane emissions. The P-values and R^2 were obtained by fitting a model including the gene and diet effects only.
