# Supplementary material for: Bovine Host Genetic Variation Influences Rumen Microbial Methane Production with Best Selection Criterion for Low Methane Emitting and Efficiently Feed Converting Hosts Based on Metagenomic Gene Abundance
Source: PLoS Genet. 2016 Feb 18;12(2):e1005846. doi: 10.1371/journal.pgen.1005846 (PMC4758630; doi:10.1371/journal.pgen.1005846)
Supplement: S4 Table — Partial least squares (PLS) estimates of effects of microbial genes characterised by the Kyoto Encyclopedia of Genes and Genomes (KEGG) database in an analysis where the PLS factors explained 80.6% of the variation of model effects and 88.3% of the variation in feed conversion ratio (kg feed intake/kg growth). (PDF) [file pgen.1005846.s009.pdf]

Table S4. Microbial genes associated with feed conversion efficiency. Partial least squares (PLS) estimates of effects of microbial genes characterised by the Kyoto Encyclopedia of Genes and Genomes (KEGG) database in an analysis where the PLS factors explained 80.6% of the variation in model effects and 88.3% of the variation in feed conversion ratio (kg feed intake/kg growth).

**KEGG-ID, breed, diet**	**Description**	**Estimate**	**VIP**	**P-value**	**R^2**
Breed AA	Aberdeen Angus	-0.12465	1.777	0.361	0.139
Breed LIM	Limousin	0.12465	1.777	0.361	0.139
K02008	cobalt/nickel transport system permease protein	0.10440	1.542	0.030	0.798
K00766	anthranilate phosphoribosyltransferase [EC:2.4.2.18]	0.08849	1.346	0.002	0.950
K02377	GDP-L-fucose synthase [EC:1.1.1.271]	-0.07758	1.297	0.004	0.924
K02907	large subunit ribosomal protein L30	0.05671	1.149	0.074	0.693
K00941	hydroxymethylpyrimidine/phosphomethylpyrimidine kinase [EC:2.7.1.49 2.7.4.7]	0.05850	1.117	0.046	0.753
K01195	beta-glucuronidase [EC:3.2.1.31]	-0.06401	1.111	0.090	0.664
K03694	ATP-dependent Clp protease ATP-binding subunit ClpA	-0.07025	1.079	0.066	0.707
K07588	LAO/AO transport system kinase [EC:2.7.-.-]	-0.06256	1.068	0.094	0.656
K00179	indolepyruvate ferredoxin oxidoreductase, alpha subunit [EC:1.2.7.8]	-0.07399	1.065	0.099	0.650
K00375	GntR family transcriptional regulator / MocR family aminotransferase	0.02972	0.959	0.041	0.765
K01876	aspartyl-tRNA synthetase [EC:6.1.1.12]	-0.05720	0.951	0.004	0.926
K06921	Putative ATPase	0.03796	0.951	0.052	0.739
K00075	UDP-N-acetylmuramate dehydrogenase [EC:1.1.1.158]	-0.01437	0.948	0.093	0.658
K01493	dCMP deaminase [EC:3.5.4.12]	0.02527	0.946	0.038	0.773
K00868	pyridoxine kinase [EC:2.7.1.35]	0.02290	0.942	0.045	0.756
K07139	Putative Fe-S oxidoreductase	-0.01605	0.937	0.065	0.710
K02006	cobalt/nickel transport system ATP-binding protein	0.03217	0.934	0.079	0.682
K00394	adenylylsulfate reductase, subunit A [EC:1.8.99.2]	0.02695	0.932	0.068	0.703
K01814	phosphoribosylformimino-5-aminoimidazole carboxamide ribotide isomerase [EC:5.3.1.16]	0.03485	0.928	0.093	0.659

Continued on next page

K03657	DNA helicase II / ATP-dependent DNA helicase PcrA [EC:3.6.4.12]	-0.00217	0.925	0.071	0.698
K01784	UDP-glucose 4-epimerase [EC:5.1.3.2]	-0.02960	0.925	0.067	0.707
K09811	cell division transport system permease protein	-0.00675	0.919	0.053	0.736
K04517	prephenate dehydrogenase [EC:1.3.1.12]	0.03268	0.919	0.075	0.690
K03581	exodeoxyribonuclease V alpha subunit [EC:3.1.11.5]	0.01700	0.917	0.051	0.739
K11752	diaminohydroxyphosphoribosylaminopyrimidine deaminase / 5-amino-6-(5-phosphoribosylamino)uracil reductase [EC:3.5.4.26 1.1.1.193]	-0.01062	0.916	0.088	0.667
K01269	aminopeptidase [EC:3.4.11.-]	0.01317	0.916	0.056	0.728
K02313	chromosomal replication initiator protein	-0.00515	0.915	0.084	0.673
K03426	NAD+ diphosphatase [EC:3.6.1.22]	0.02591	0.915	0.087	0.669
K00773	queuine tRNA-ribosyltransferase [EC:2.4.2.29]	-0.02257	0.910	0.073	0.694
K13542	uroporphyrinogen III methyltransferase / synthase [EC:2.1.1.107 4.2.1.75]	0.02152	0.910	0.064	0.713
K03631	DNA repair protein RecN (Recombination protein N)	0.01067	0.905	0.048	0.747
K08602	oligoendopeptidase F [EC:3.4.24.-]	0.01777	0.904	0.083	0.676
K00956	sulfate adenylyltransferase subunit 1 [EC:2.7.7.4]	0.00927	0.902	0.090	0.663
K01924	UDP-N-acetylmuramate--alanine ligase [EC:6.3.2.8]	-0.01173	0.901	0.087	0.668
K00974	tRNA nucleotidyltransferase (CCA-adding enzyme) [EC:2.7.7.72 3.1.3.- 3.1.4.-]	0.01214	0.886	0.092	0.661
K01818	L-fucose isomerase [EC:5.3.1.25]	-0.04998	0.884	0.067	0.707
K01129	dGTPase [EC:3.1.5.1]	-0.01655	0.881	0.043	0.760
K08483	phosphotransferase system, enzyme I, PtsI [EC:2.7.3.9]	0.00303	0.880	0.077	0.686
K03458	nucleobase:cation symporter-2, NCS2 family	0.01331	0.873	0.090	0.663
K03111	single-strand DNA-binding protein	0.02467	0.869	0.010	0.883
K00278	L-aspartate oxidase [EC:1.4.3.16]	-0.02361	0.868	0.082	0.678
K03501	glucose inhibited division protein B [EC:2.1.-.-]	-0.02020	0.862	0.066	0.707
K02343	DNA polymerase III subunit gamma/tau [EC:2.7.7.7]	0.02796	0.850	0.008	0.891
K01104	protein-tyrosine phosphatase [EC:3.1.3.48]	0.01502	0.843	0.025	0.815

Continued on next page

Table S4 – Continued from previous page

K07082	UPF0755 protein	-0.00521	0.837	0.076	0.687
K01928	UDP-N-acetylmuramoylalanyl-D-glutamate--2,6-diaminopimelate ligase [EC:6.3.2.13]	-0.01444	0.830	0.023	0.820
Diet CON	Concentrate based diet	-0.05925	0.829	0.429	0.107
Diet FOR	Forage based diet	0.05925	0.829	0.429	0.107
K03615	electron transport complex protein RnfC	0.03462	0.819	0.049	0.745
K01613	phosphatidylserine decarboxylase [EC:4.1.1.65]	0.01464	0.799	0.009	0.888
K01358	ATP-dependent Clp protease, protease subunit [EC:3.4.21.92]	-0.03591	0.731	0.020	0.833

Two factors were significant in the partial least squares analysis. Without considering diet and breed in the model, , the microbial genes explained 83.9% of the variation of model effects and 85.5% of the variation in feed conversion ratio. The PLS estimates were based on predictors and responses centered and scaled to have mean 0 and standard deviation 1. Variable Importance for Projection (VIP) statistic of Wold, which summarizes the contribution of a variable maker to the model were used to identify the significant microbial genes associated with feed conversion ratio. The P-value and R^2 were obtained by fitting a model including the gene and diet effects only.
